# Head in the clouds: two new microendemic tepui-summit species of *Stefania* (Anura: Hemiphractidae)

**DOI:** 10.1186/s40851-024-00237-w

**Published:** 2024-08-01

**Authors:** Philippe J.R. Kok

**Affiliations:** 1 Department of Ecology and Vertebrate Zoology, Faculty of Biology and Environmental Protection, University of Łódź, 12/16 Banacha Str, Łódź, 90-237 Poland; 2 Department of Life Sciences, The Natural History Museum, Cromwell Road, London, SW7 5BD UK

**Keywords:** Homoplasy, Morphology, Osteology, Pantepui, Systematics, Taxonomy

## Abstract

In addition to the type locality (the summit of Aprada-tepui, Bolívar State of Venezuela), the distribution of the egg-brooding frog *Stefania satelles* was long thought to include several isolated tabletop mountain (tepui) summits surrounding the large Chimantá Massif in Bolívar State (hence the Latin name “*satelles*”). However, multilocus molecular phylogenetic analyses have revealed that this taxon includes several undescribed morphologically cryptic species, and that *S. satelles* should be restricted to its type locality. Two tepui-summit species confused under that name in the literature remain to be named, and the present paper aims at describing these populations previously referred to as *Stefania* sp. 3 and *S*. sp. 5. *Stefania* sp. 3 is only known from the small summit of Angasima-tepui, while *S*. sp. 5 is only reported from the small summit of Upuigma-tepui, both mountains being located south of the Chimantá Massif. These new, phylogenetically distinct species are described based on external morphology and osteology and in comparison to close relatives in the *S. ginesi* clade, which consists exclusively of tepui summit species. Both new species have highly restricted geographic ranges (less than 3 km^2^) and should be listed as Critically Endangered according to IUCN criteria.

## Introduction

**Fig. 1:**
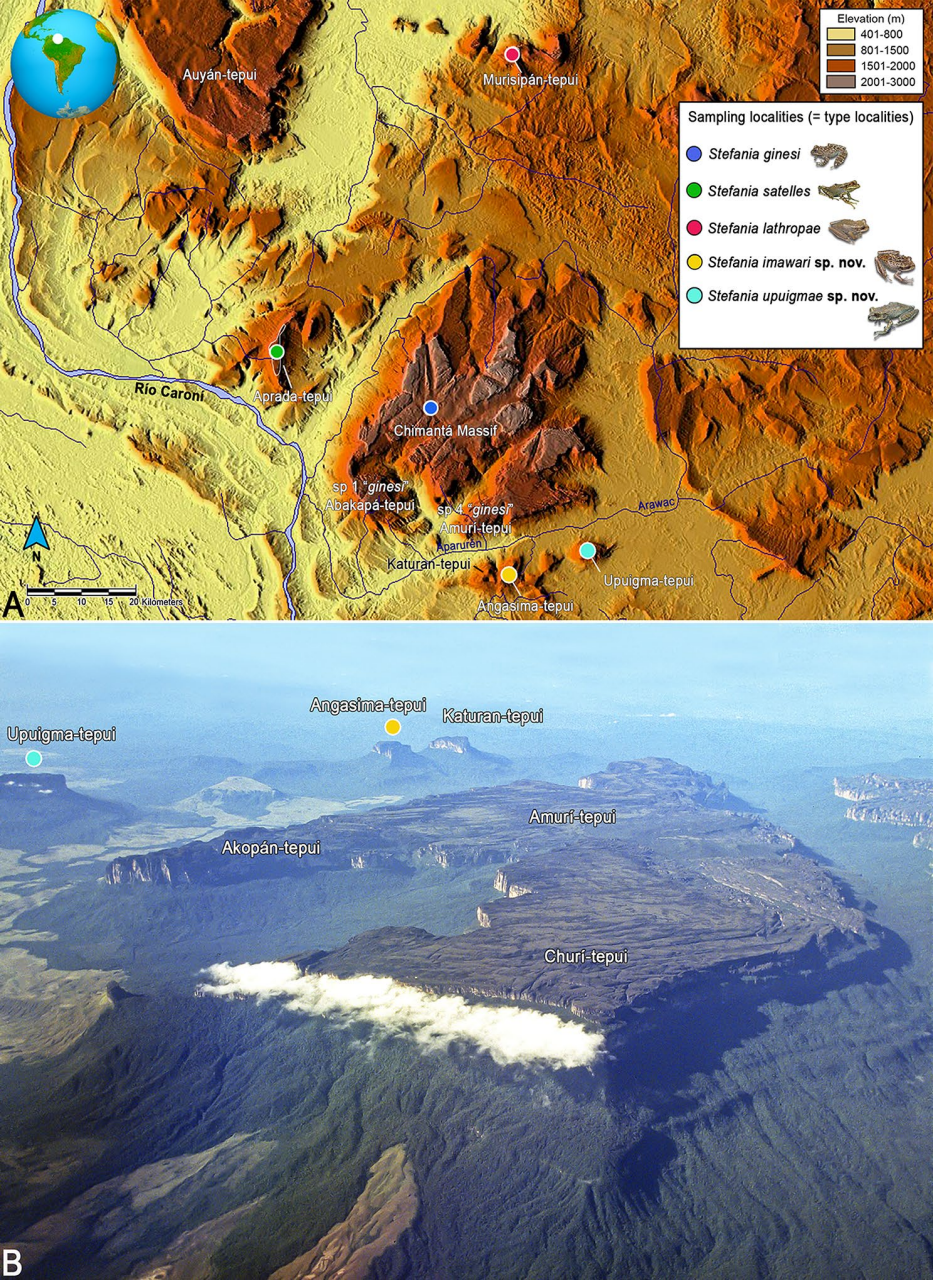
Distribution of species in the *Stefania ginesi* clade (**A**). Distribution map of the *Stefania ginesi* clade as currently understood. Locality data are based on specimens examined (see Appendix) and literature records [8, 14, 15, 34]. Inset photos by the author. (**B**). Aerial photograph of the southern part of the Chimantá massif, taken facing southwest showing the distribution of *Stefania imawari* sp. nov. and *S. upuigmae* sp. nov. Photo by C. Brewer-Carías

**Fig. 2:**
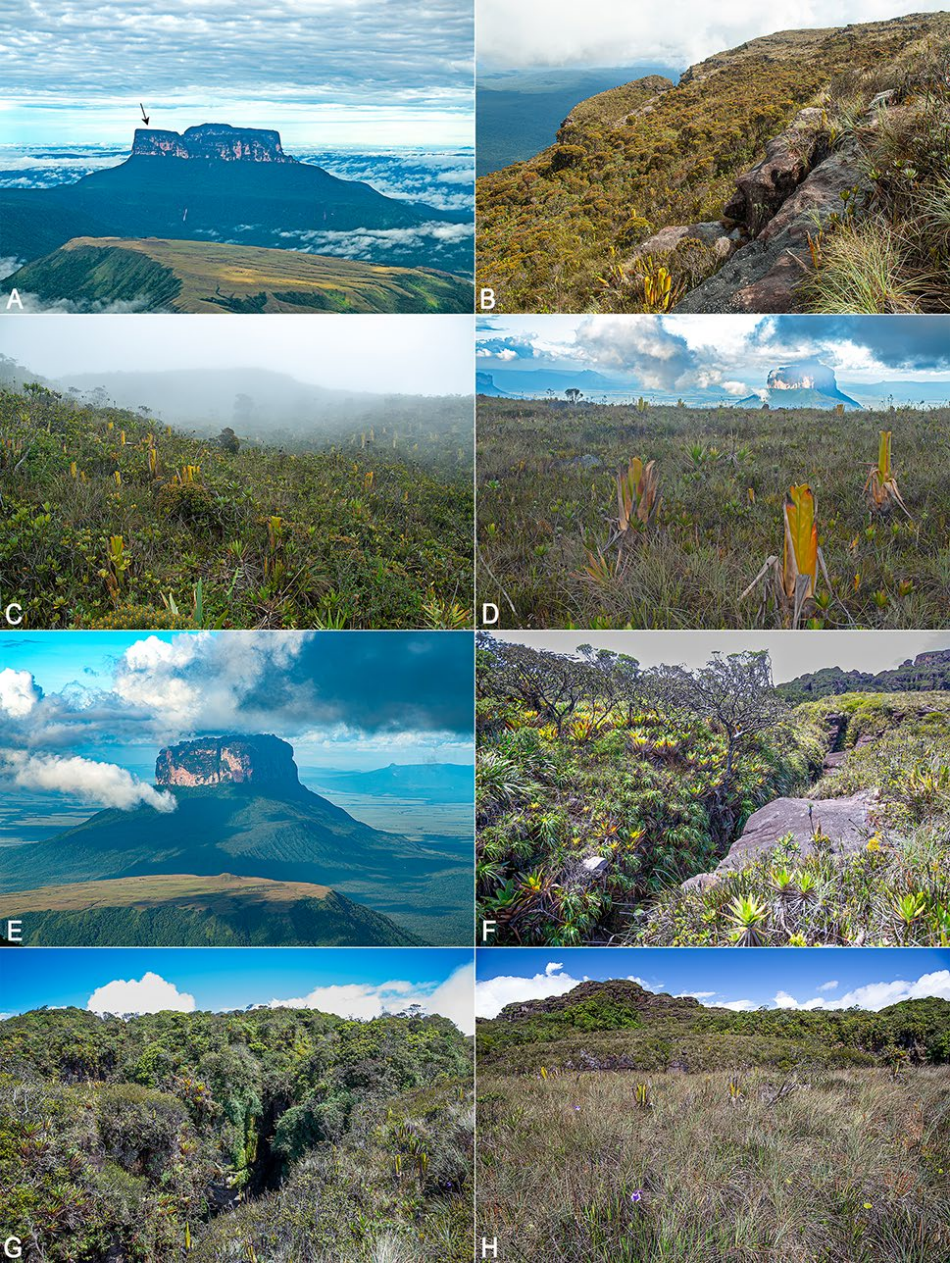
Type localities and habitats of *Stefania imawari* sp. nov. and *S. upuigmae* sp. nov. (**A**). Angasima-tepui as seen from the summit of Upuigma-tepui; black arrow indicates the exact type-locality of *S. imawari*. (**B–D**). Vegetation on the summit of Angasima-tepui. (**E**). Upuigma-tepui as seen from the summit of Angasima-tepui. (**F–H**). Vegetation on the summit of Upuigma-tepui. Photos by the author

The genus *Stefania* belongs to the frog family Hemiphractidae, a Neotropical lineage currently comprising six genera and more than 120 species [1]. Hemiphractids are mostly characterised by a unique breeding behaviour consisting of females carrying eggs on their back, either exposed or enclosed in dorsal pouches [2]. In *Stefania* the exposed eggs are glued to the female’s dorsum [3] by fluids secreted by the female during amplexus [4] and develop into froglets on the female’s back with no free-swimming tadpole stage [2]. This behaviour is a competitive evolutionary advantage for survival in harsh environments, such as nutrient-poor mountaintops [5]. The number of froglets carried by a single female can reach up to 30 in *S. evansi*, the largest known species in the genus with a maximum snout-vent length (SVL) of 97.5 mm [6, 7]. The genus *Stefania* is considered a Pantepui (western Guiana Shield highlands) endemic [8–10], with most species inhabiting the slopes or summits of sandstone tabletop mountains (locally known as tepuis), although one species (*S. evansi*) has dispersed down to ca. 100 m above sea level (asl) in the neighbouring lowland rainforests of western Guyana [10]. Due to their restricted elevational ranges, most microendemic *Stefania* species are likely threatened by ongoing and projected climate change (e.g., [11]), a common fate of Pantepui amphibian lineages (e.g., [12]). Several morphologically cryptic species have been reported in the genus [10, 13, 14], and the present work aims at describing two *Stefania* populations long confused with *S. satelles*, and previously referred to as *Stefania* sp. 3 and *S*. sp. 5 [10, 15]. Both species are seemingly single tepui summit endemics, with *S*. sp. 3 only known from the small summit of Angasima-tepui (summit surface ca. 2.5 km^2^), and *S*. sp. 5 only reported from the small summit of Upuigma-tepui (summit surface ca. 2.3 km^2^), both mountains being located south of the Chimantá Massif in the Bolívar state of southern Venezuela (Fig. 1). The summits of Angasima-tepui and Upuigma-tepui are presently strongly isolated from each other, and from other summits of the Chimantá Massif, by large stretches of upland rainforest and savannah and by substantial watercourses, such as the rivers Aparurén and Arawac (Figs. 1 and 2). The two new species are allopatric, and morphologically and phylogenetically distinct from other *Stefania* species in the *ginesi* clade, which exclusively consists of tepui summit species found at elevations between 2,115 and 2,580 m. The *S. ginesi* clade as currently recognized [10, 13, 15; this paper] is endemic to the Chimantá Massif and a few peripheral tepuis in southeastern Venezuela.

## Materials and methods

### Specimens examined

Specimens examined for this study are listed in the Appendix and in the new species descriptions below. Specimens of the two new species were registered in the collections of the Royal Belgian Institute of Natural Sciences (IRSNB; Brussels, Belgium) under the name *Stefania satelles* for more than a decade, but have now been assigned new museum numbers according to their new status as type specimens.

### Morphometrics and morphological data

Morphological examinations and measurements of the type series were performed under a Leica M205C stereomicroscope. Morphometric data were taken from the preserved specimens to the nearest 0.01 mm (rounded to 0.1 mm) with digital callipers (MarCal 16 EWRi). Morphological comparisons are based on examination of museum specimens (see Appendix) and published descriptions [8, 15–28]. Description of external morphological characters mostly follows Kok and Kalamandeen [29] and Duellman [2]. Definition, diagnosis, and description of the holotypes mostly follow the scheme of MacCulloch and Lathrop [22], with amendments as provided in Kok [15, 28], for ease of comparison.

### Micro-computed tomography (µCT) scanning, 3D reconstructions, and osteology

Specimens were µCT-scanned at the Natural History Museum’s (NHM, London) CT Lab facility using a Nikon HMX225. All osteological images were exported from the virtual 3D models, which were reconstructed and segmented using VGStudio MAX version 2.1. Three-dimensional mesh files of full bodies (including new full-body µCT-scans of the holotype and one paratype of *S. lathropae*) and crania have been deposited on the MorphoSource platform at the following URL: https://www.morphosource.org/projects/000642378. Osteological terminology follows Trueb [30] and Duellman [2], and osteological description follows the scheme of Kok [15, 28]. The term “exostosis” is used for “dermal sculpturing” (e.g., [31]). Estimation of the degree of contact between bony structures followed Kok et al. [32], i.e., contacting/in contact = contact between structures with a visible suture line, and fused = contact between structures with a suture line being barely visible or absent. Osteological comparisons with other species in the *Stefani ginesi* clade were based on Kok [15, 28] and on examination of newly acquired µCT data of *S. lathropae*.

## Results

**Fig. 3:**
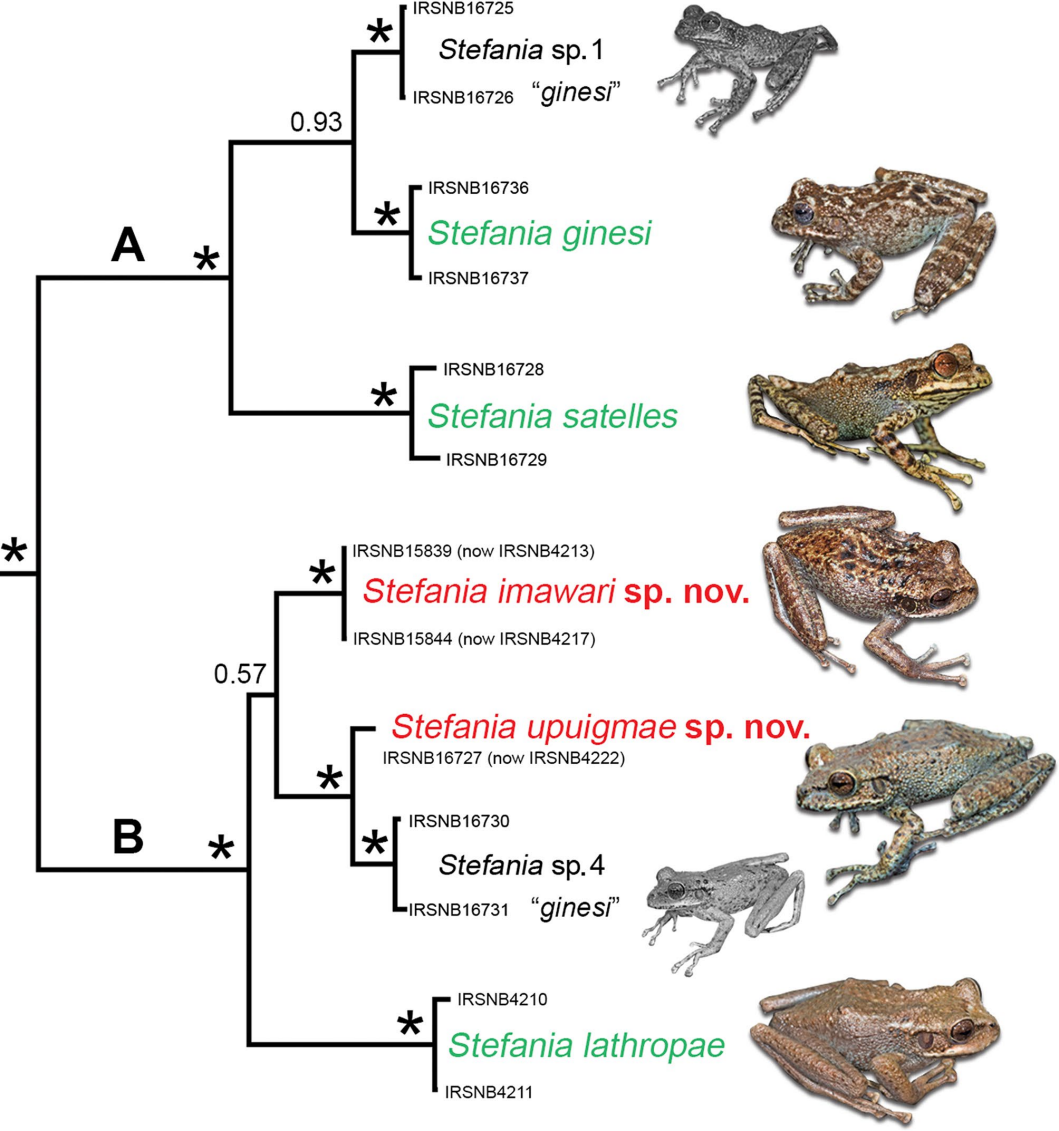
Phylogenetic relationships of the *Stefania ginesi* clade modified from [14], based on 2,359 base pairs of nuclear and mitochondrial DNA (Bayesian statistical supports are provided at nodes, asterisks indicate values > 95%). *Stefania* sp. 1 and *S*. sp. 4 remain undescribed and were both confused with *S. ginesi*. Photos by the author

Both new species are placed in the *Stefania ginesi* clade based on molecular data (see [10, 14]; Fig. 3) and morphological features. Members of the *S. ginesi* clade are exclusively found on tepui summits and are mainly characterized by (1) small to medium size (< 65 mm SVL); (2) head about as wide as long; (3) dorsal skin tuberculate; (4) canthus rostralis tuberculate; (5) low parietal crests; (6) toes basally webbed; (7) iris generally unicolour—or faintly bicolour—copper, dark brown, or greyish blue in life; (8) males with nuptial excrescences on thumb. Such a combination of characters is not found in any other known *Stefania* clade.

Although these data are only exploratory and should be interpreted cautiously (see [33]), the molecular divergence between the two new species is 3.2% in the barcoding fragment of 16S rRNA (see [15]; summarised in Table 1). Molecular divergence with *S. lathropae* (the closest described relative) varies from 3.2 to 3.4% (*S. imawari* sp. nov.) and from 4.5 to 4.7% (*S. upuigmae* sp. nov.) (Table 1). Intraspecific genetic divergences in species of the *S. ginesi* clade vary from 0 to 0.2%, which is identical to the intraspecific divergences observed in species of the *S. riveroi* clade, including across allopatric populations [28].

**Table 1:** Genetic distances in the barcoding fragment of 16S rRNA (533 base pairs) within the *Stefania ginesi* clade. (modified from Kok [15])

		1	2	3	4	5	6	7	8	9	10	11	12	13
1	*Stefania ginesi* (IRSNB16736)													
2	*Stefania ginesi* (IRSNB16739)	0.000												
3	*Stefania satelles* (IRSNB16728)	0.053	0.053											
4	*Stefania satelles* (IRSNB16729)	0.051	0.051	0.002										
5	*Stefania lathropae* (IRSNB4210)	0.077	0.077	0.086	0.085									
6	*Stefania lathropae* (IRSNB4211)	0.078	0.078	0.087	0.085	0.002								
7	*Stefania imawari *sp. nov. (IRSNB4213)	0.081	0.081	0.081	0.079	0.034	0.032							
8	*Stefania imawari*s p. nov. (IRSNB4217)	0.081	0.081	0.081	0.079	0.034	0.032	0.000						
9	*Stefania* sp. 1 (IRSNB16725)	0.017	0.017	0.039	0.038	0.083	0.083	0.077	0.077					
10	*Stefania* sp. 1 (IRSNB16726)	0.017	0.017	0.039	0.038	0.083	0.083	0.077	0.077	0.000				
11	*Stefania* sp. 4 (IRSNB16730)	0.079	0.079	0.085	0.083	0.043	0.042	0.024	0.024	0.075	0.075			
12	*Stefania* sp. 4 (IRSNB16731)	0.084	0.084	0.090	0.090	0.046	0.044	0.027	0.027	0.080	0.080	0.000		
13	*Stefania upuigmae *sp. nov. (IRSNB4222)	0.085	0.085	0.092	0.090	0.047	0.045	**0.032**	**0.032**	0.081	0.081	0.008	0.006	

### *Stefania imawari* sp. nov.

*Stefania satelles* [in part] Señaris et al., 1997 [8]: 33–37.

*Stefania satelles* [in part] Gorzula and Señaris, 1999 [34]: 47.

*Stefania satelles* [in part] McDiarmid and Donnelly, 2005 [9]: 513, 521.

*Stefania satelles* Kok et al., 2012 [13]: Supplemental Information.

*Stefania satelles* [in part] Kok et al., 2016 [14]: 6.

*Stefania* sp. 3 Kok et al., 2017 [10]: 175–176.

**ZooBank registration**. urn: lsid: zoobank.org: act:6FBDF6A6-F65D-4D6A-82BD-6021071B16CB.

**Fig. 4:**
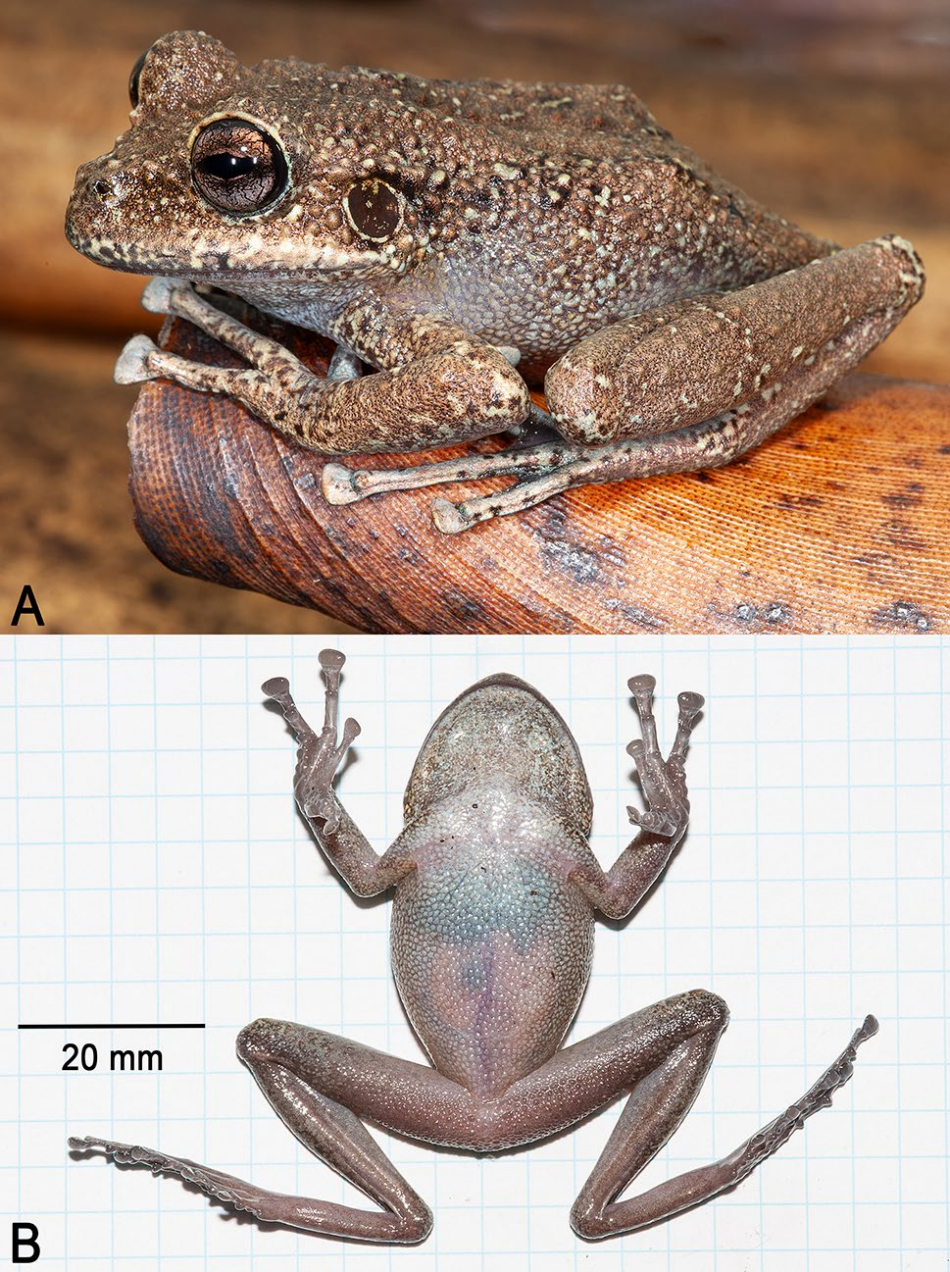
Holotype of *Stefania imawari* sp. nov. (IRSNB 4221, 48.5 mm SVL) (**A**). Dorsolateral view in life. (**B**). Ventral view of the specimen freshly euthanized. Photos by the author

**Fig. 5:**
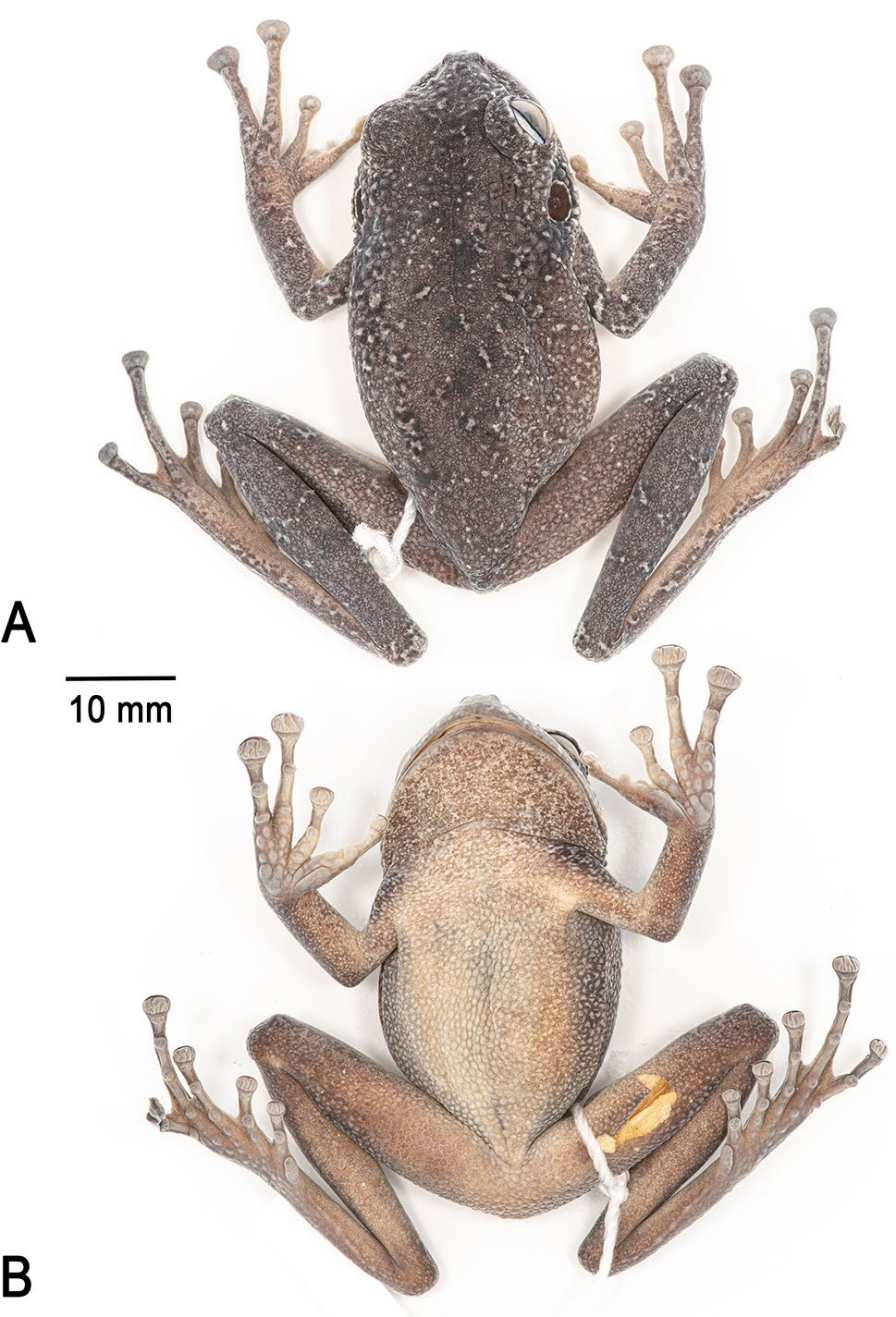
Holotype of *Stefania imawari* sp. nov. (IRSNB 4221, female, 48.5 mm SVL) after 13 years in ethanol preservative. (**A**). Dorsal view. (**B**). Ventral view. Photos by the author

**Fig. 6:**
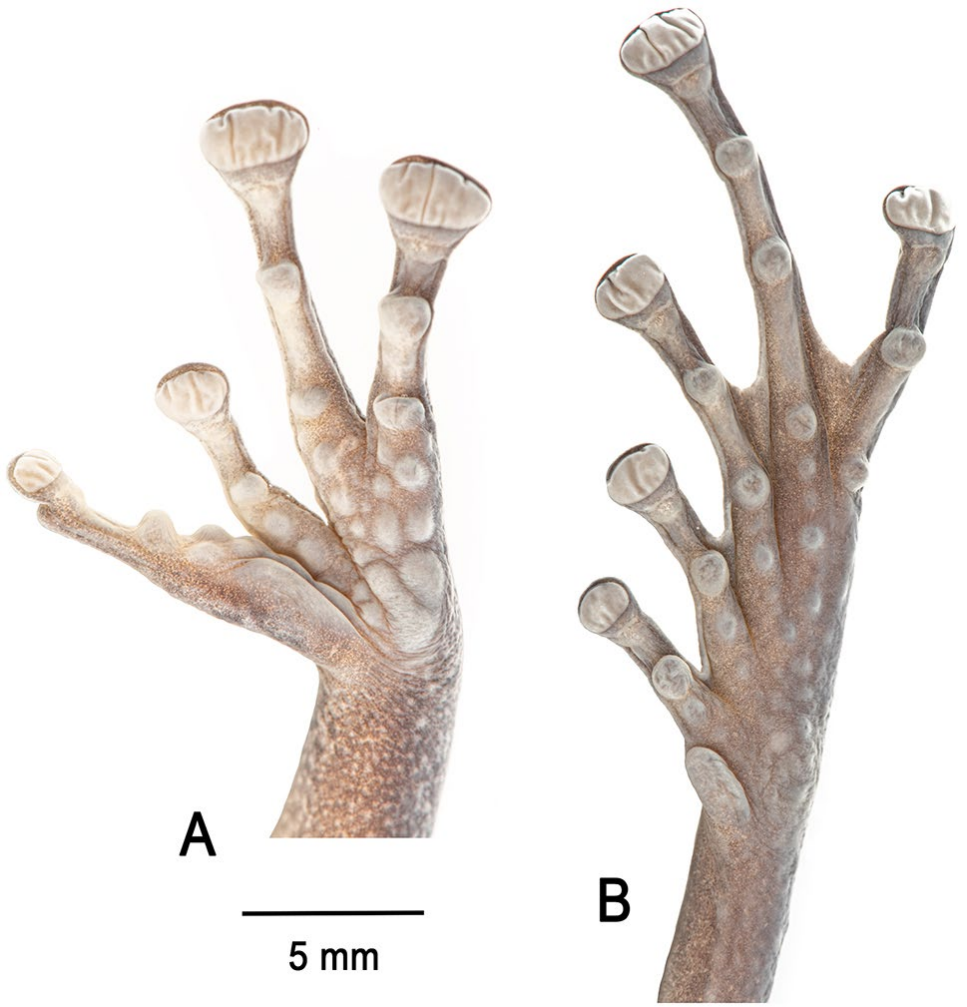
Palm (**A**) and sole (**B**) of the preserved holotype of *Stefania imawari* sp. nov. (IRSNB 4221, female, 48.5 mm SVL). Photos by the author

**Holotype**. IRSNB 4221 (field number PK3605, Figs. 4, 5 and 6), an adult female collected by Philippe J. R. Kok, 12 May 2011 at 16h20, summit of Angasima-tepui, Bolívar State, Venezuela (05°02′35″N, 62°04′52″W; 2,154 m elevation).

**Fig. 7:**
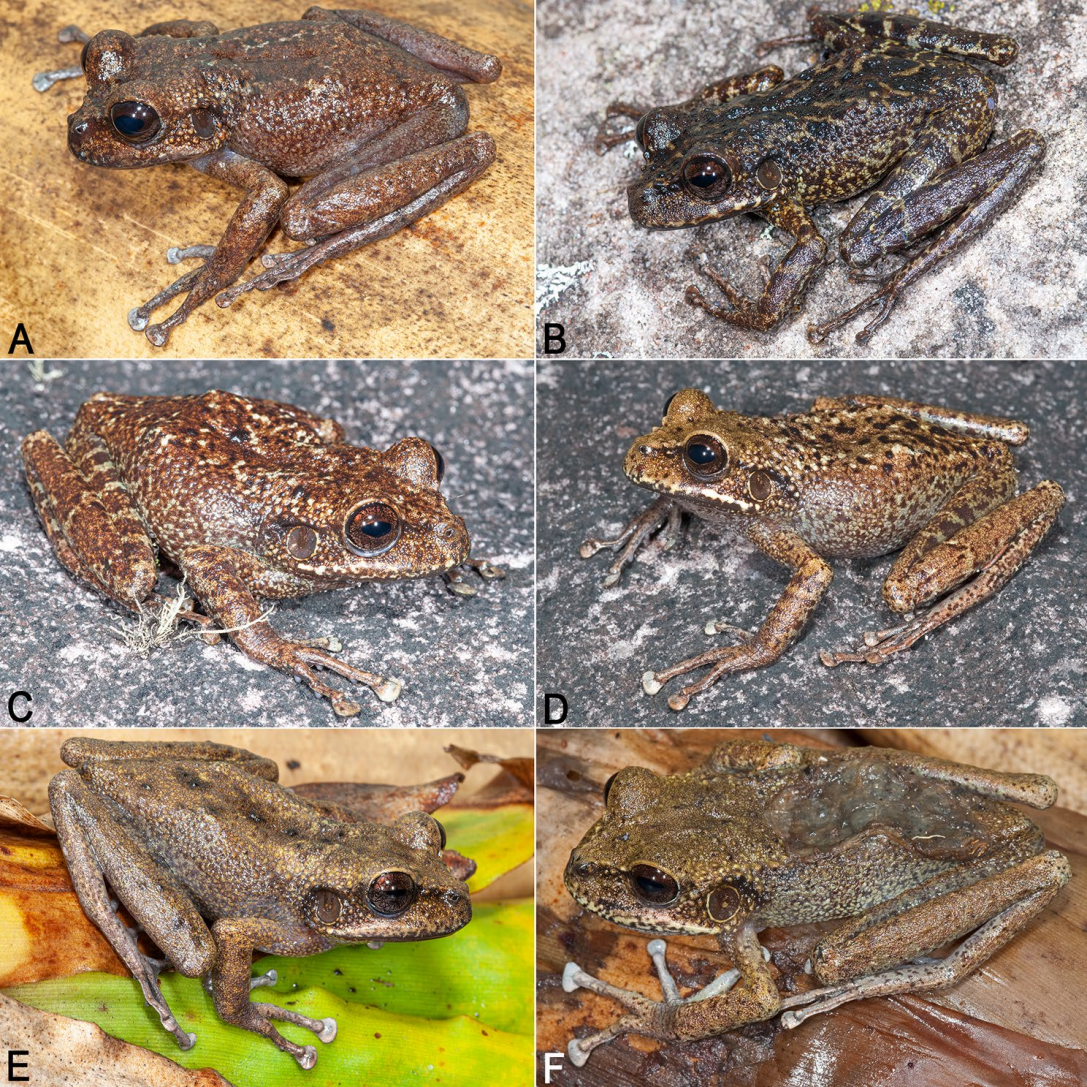
Main colour pattern variation in *Stefania imawari* sp. nov. (**A**). IRSNB 4218, male, 48.9 mm SVL. (**B**). IRSNB 4213, immature female, 38.0 mm SVL. (**C**). IRSNB 4215, male, 44.1 mm SVL. (**D**). IRSNB 4214, male, 44.4 mm SVL. (**E**). IRSNB 4219, male, 44.6 mm SVL. (**F**). IRSNB 4220, female, 55.4 mm SVL. Photos by the author

**Paratopotypes** (*n* = 8; Fig. 7). IRSNB 4214 (adult male; field number PK3602) collected by Philippe J.R. Kok, 11 May 2011 at 19h20, summit of Angasima-tepui, Bolívar State, Venezuela (5°02′36″N, 62°04′51″W; 2,122 m elevation); IRSNB 4215 (adult male; field number PK3603) collected by Philippe J.R. Kok, 11 May 2011 at 19h25, summit of Angasima-tepui, Bolívar State, Venezuela (5°02′36″N, 62°04′51″W; 2,122 m elevation); IRSNB 4216 (adult male; field number PK3604) collected by Philippe J.R. Kok, 11 May 2011 at 19h00, summit of Angasima-tepui, Bolívar State, Venezuela (5°02′36″N, 62°04′51″W; 2,122 m elevation); IRSNB 4218 (adult male; field number PK3608) collected by Philippe J.R. Kok, 12 May 2011 at 20h10, summit of Angasima-tepui, Bolívar State, Venezuela (5°02′36″N, 62°04′51″W; 2,122 m elevation); IRSNB 4219 (adult male; field number PK3609) collected by Philippe J.R. Kok, 13 May 2011 at 6h00, summit of Angasima-tepui, Bolívar State, Venezuela (5°02′36″N, 62°04′51″W; 2,122 m elevation); IRSNB 4213 (immature female; field number PK3600) collected by Philippe J.R. Kok, 11 May 2011 at 8h40, summit of Angasima-tepui, Bolívar State, Venezuela (5°02′36″N, 62°04′51″W; 2,122 m elevation); IRSNB 4217 (adult female; field number PK3607) collected by Philippe J.R. Kok, 12 May 2011 at 20h00, summit of Angasima-tepui, Bolívar State, Venezuela (5°02′36″N, 62°04′51″W; 2,122 m elevation); IRSNB 4220 (adult female; field number PK3611) collected by Philippe J.R. Kok, 13 May 2011 at 19h00, summit of Angasima-tepui, Bolívar State, Venezuela (5°02′36″N, 62°04′51″W; 2,122 m elevation).

**Etymology**. The specific epithet *imawari* is a noun used in apposition and refers to the malicious spirits that inhabit the tepuis, according to Pemón traditional beliefs (Arturo Berti, pers. comm.). The Pemón are indigenous people living in the southeast region of Venezuela, including the area surrounding the type locality.

**Definition and diagnosis**. *Stefania imawari* sp. nov. is characterized by the following morphological characters, the combination of which distinguishes it from all known congeners: (1) a small species of *Stefania*, max. SVL in preserved adult females 55.4 mm (*n* = 3), 48.9 mm in adult preserved males (*n* = 5); (2) head not distinctly longer than wide, about as wide as long; (3) canthus rostralis angular, slightly concave in dorsal view (almost straight), tuberculate, 1–3 distinctly enlarged canthal tubercles that project dorsolaterally, ill-defined canthal stripe present in life; (4) loreal region with a few low tubercles; (5) upper eyelid smooth to tuberculate, none of the tubercles distinctly enlarged; (6) frontoparietal ridges present, but low and barely visible (both in life and in preservative); (7) frontoparietal crests barely developed (on cranium); (8) constriction of the frontoparietal bones at the level of the anterior epiotic eminence; (9) low, sometimes extensive, exostosis on the cranium; (10) premaxillae slightly projecting posteriorly in lateral view; 11) posterodorsal projection of maxilla absent or highly reduced; 12) maxillary process of the nasal not in contact with the maxilla; 13) horizontal length of tympanum more than 50% horizontal length of eye in both sexes; 14) vomerine teeth 3–7; 15) toes basally webbed, no significant difference in toe webbing between sexes; 16) dorsal skin (in life) tuberculate; 17) ventral skin (in life) granular; 18) absence of a conspicuous/enlarged outer tarsal tubercle; 19) two large, creamy brown, medially fused, nuptial excrescences present on the thumb in males; 20) absence of multiple conspicuous dark brown bars on flanks and lips, absence of white dorsolateral stripes (in life); 21) in living adults, iris faintly bicolour, dark brown/dark copper, with extensive dark brown reticulations; a faint blueish-grey tint may be present depending on the luminosity.

*Stefania imawari* sp. nov. is further distinguished from the other described species in the *S. ginesi* clade as follows:

From *Stefania ginesi* mostly by the colour of the iris (greyish blue in *S. ginesi* vs. dark brown/dark copper in *S. imawari* sp. nov., although in a few specimens the iris may have a faint blueish grey tint, depending on the luminosity); by the size and number of tubercles on the dorsum, flanks, and arms/legs (distinctly larger and more numerous in *S. ginesi*); by a more angular canthus rostralis (less defined in *S. ginesi*); and by the size of tubercles on the canthus rostralis and upper eyelid (larger in *S. ginesi*).

From *Stefania lathropae* mostly by the absence of white dorsolateral stripes (always present, even if sometimes short, in *S. lathropae*); by the supratympanic fold barely obscuring the upper margin of the tympanum (vs. distinctly obscuring the upper margin of the tympanum in *S. lathropae*); and by the colour of the iris (unicolour, copper in *S. lathropae* vs. faintly bicolour, dark brown/dark copper in *S. imawari* sp. nov.). Notable potential osteological diagnostic characters are the length of the postchoanal process of the vomer (long and reaching the neopalatine in *S. lathropae* vs. short in *S. imawari* sp. nov.); and the length of the dorsal process on the anterior ramus of the pterygoid projecting towards the zygomatic ramus of the squamosal (longer in *S. lathropae*).

From *Stefania satelles* mostly by the osteology of the frontoparietal crests (moderately developed and distinctly projecting dorsolaterally in *S. satelles* vs. barely developed in *S. imawari* sp. nov.); and by the colour of the iris (unicolour, copper in *S. satelles* vs. faintly bicolour, dark brown/dark copper in *S. imawari* sp. nov.).

**Description of the holotype**. An adult female 48.5 mm SVL (Figs. 4, 5 and 6), in good condition except for long incisions made during tissue sampling (left thigh) and during the examination of internal organs (left side of the abdomen). Head slightly wider than long; head wider than neck. Snout rounded in dorsal and lateral views, slightly mucronate in dorsal view, ca. 1.3 times the horizontal length of eye. Eye–nostril distance ca. 0.9 times the horizontal length of eye, more than three times the distance between the nostril and the tip of the snout. Canthus rostralis angular, slightly concave in dorsal view, sloping; lips flared. Nostrils protuberant, directed anterolaterally. Internarial distance ca. 50% of interorbital distance. Internarial region concave. Interorbital distance slightly longer than upper eyelid width. Frontoparietal ridges low and barely visible. Temporal region slightly bulged. Tympanum distinct, large, round, directed posterolaterally, 64% of horizontal length of eye, separated from eye by ca. 59% of horizontal length of eye. Supratympanic fold prominent, extending from posterior corner of eye to above insertion of forelimb, barely obscuring upper margin of tympanum.

Choanae small and oval. Vomerine processes transverse between choanae, not in contact, larger than choanae, each bearing ca. seven teeth. Tongue large, ovoid. Palpebral membrane not reticulated, upper rim with a light brown band.

Dorsal skin tuberculate, venter and posterior surface of thighs granular; a few low tubercles on shank and lower arm. Upper eyelid tuberculate, none of these tubercles is distinctly enlarged. Loreal region with a few low tubercles. Tympanic region strongly tuberculate. Canthus rostralis tuberculate, 1–2 canthal tubercles distinctly enlarged and projecting dorsolaterally. Cloacal opening directed posteriorly at upper level of thighs, presence of a short cloacal flap.

Thenar tubercle large, distinct, transversely oval; palmar tubercle distinct, about half the size of thenar tubercle. Subarticular tubercles distinct, large, projecting, and single in appearance. Supernumerary tubercles numerous, most of them large and projecting (Fig. 6). Relative finger lengths II < I < IV < III; adpressed second finger barely reaches the first finger’s disc. Fingers with lateral fringes; no distinct webbing between FIII-FIV, which are fused proximally. Finger discs large, transversely oval, more than two times wider than adjacent phalange on FIII and FIV, smallest and subequal in length on FI and FII, largest and subequal in length on FIII-IV. Largest disc width (FIV) 87% of horizontal length of tympanum.

Inner metatarsal tubercle large, oval, distinct; outer metatarsal tubercle not detectable on left foot, on right foot about 5.2 times smaller than inner, round, poorly distinct, very similar to surrounding supernumerary tubercles, but slightly darker. Subarticular tubercles single, round, distinct. Supernumerary tubercles numerous, round, distinct, most of them similar in size (Fig. 6). Relative lengths of toes I < II < III < V < IV; adpressed fifth toe distinctly longer than third. Toes basally webbed, webs tapering to lateral fringes. Webbing formula II 1 ¾–3 III 2^−^–3 ½ IV 3 ¼–2 V. Toe discs oval, wider than adjacent phalange, largest toe disc on Toe IV, ca. 81% width of largest finger disc. Heels overlapping when hindlimbs are folded at right angles to sagittal body plane.

**Colour of holotype in life**. Dorsum light to medium brown with a few creamy white/beige spots, often circled in black and often corresponding to large tubercles (Fig. 4). Upper flanks similar to dorsum, lower flanks similar to dorsum medially, but greyish brown anteriorly and posteriorly. Flanks speckled with brown, dark brown, and creamy white/beige small spots. Anterodorsal aspect of upper and lower arm light brown, with a few brown and creamy white/beige/black small spots. Anterodorsal aspect of thigh and shank light brown, with a few brown and creamy white/beige small spots. Hands and feet greyish brown speckled with small brown and creamy white/beige/black spots. Top of head and upper eyelid medium brown, similar to dorsum. Canthal stripe barely visible, dark brown; short black supratympanic stripe present; post-tympanic region dark brown. Tympanum dark brown, almost black, with a few tiny creamy white speckles; tympanum circled in creamy white. Upper lip creamy white, with some scattered melanophores more obvious and numerous anteriorly; lower lip greyish brown with a few dark brown markings. Throat and anterior thorax creamy white with medium brown speckles on throat. Venter and underside of limbs light/medium grey, with some rare dark brown mottling, especially under knee. The belly is slightly translucent (some internal organs visible). Palms and soles dark grey. Iris faintly bicolour, light copper on its upper part, light bluish grey on its lower part, with extensive dark brown reticulations. Eye mostly circled in creamy white.

**Colour of holotype in preservative**. After almost 13 years in ethanol preservative (Fig. 5), the overall colouration has faded but remains similar compared to the condition in life. The ventral face turned opaque creamy brown, and the medium brown speckling became darker and more conspicuous. Palms turned creamy brown, and soles are now dark greyish brown.

**Fig. 8:**
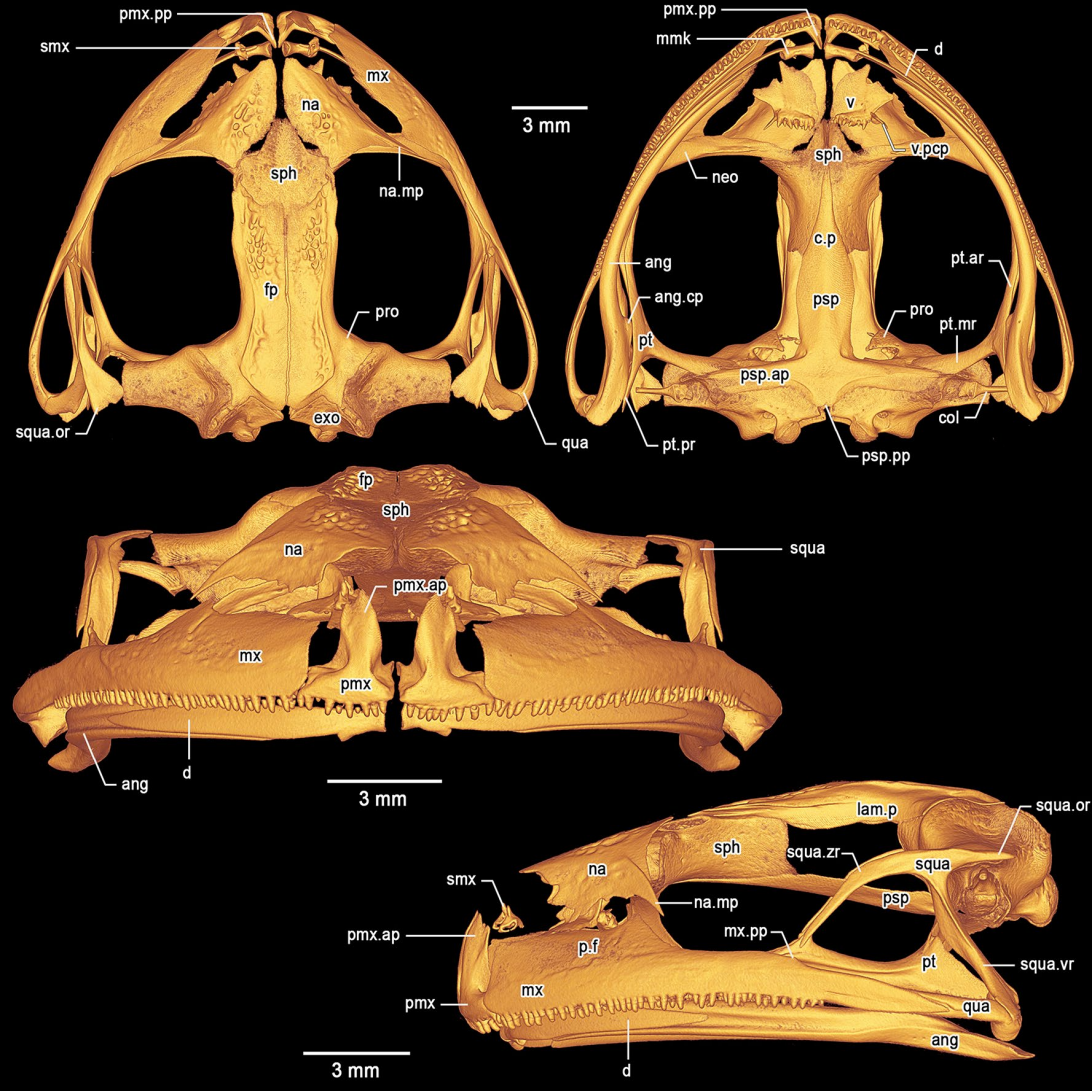
Three-dimensional model of the cranium of the female holotype of *Stefania imawari* sp. nov. (IRSNB 4221) based on µCT imagery. Top left: dorsal view. Top right: ventral view. Middle: frontal view. Bottom: left lateral view. Abbreviations: ang = angulosplenial, ang.cp = coronoid process of the angulosplenial, col = columella, c.p = cultriform process, d = dentary, exo = exoccipital, fp = frontoparietal, lam.p = lamina perpendicularis, mmk = mentomeckelian, mx = maxilla, mx.pp = posterodorsal projection of the maxilla, na = nasal, na.mp = maxillary process of the nasal, neo = neopalatine, p.f = pars facialis, pmx = premaxilla, pmx.ap = alary process of the premaxilla, pmx.lp = lateral process of the premaxilla, pmx.pp = palatine process of the premaxilla, pro = prootic, psp = parasphenoid, psp.ar = alary process of the parasphenoid, psp.pp = posteromedial process of the parasphenoid, pt = pterygoid, pt.ar = anterior ramus of the pterygoid, pt.mr = medial ramus of the pterygoid, pt.pr = posterior ramus of the pterygoid, qua = quadratojugal, smx = septomaxilla, sph = sphenethmoid, squa = squamosal, squa.or = otic ramus of the squamosal, squa.vr = ventral ramus of the squamosal, squa.zr = zygomatic ramus of the squamosal, v = vomer, v.pcp = postchoanal process of the vomer

**Osteology of holotype**. *Cranium* (Fig. 8). The skull is large, wider than long (greatest width ca. 127% of medial length). The braincase appears mostly ossified; the sphenethmoid complex is not dorsally invested by the nasals. The exoccipital-prootic complex is well ossified and laterally barely overlapped by the otic ramus of the squamosal. The paired septomaxillae are well developed and lie dorsal to the palatine process and posterolaterally to the articulation between the maxilla and premaxilla. The columellae (stapes) are ossified, formed by the synostotic fusion of the long, thin pars media plectri (stylus) and the pars interna plectri (baseplate), which is curved.

*Dorsal investing bones*. The nasals are broad, slightly exostosed, not contacting medially. The posteromedial margins of the nasals do not invest the sphenethmoid. The maxillary process of the nasal is long, acuminate, slightly curved in dorsal view (concave), not in contact with the maxilla. The frontoparietals are slightly exostosed and completely roof the central braincase from the anterior level of the orbit to the level of the tectum synoticum posteriorly. Lamina perpendicularis poorly developed along the anterior orbital margin of the frontoparietal, expanding posteriorly. Posteriorly, the frontoparietal slightly expands dorsolaterally to form a very low frontoparietal crest, which bears low exostosis.

*Ventral investing and palatal bones*. The parasphenoid is nib-shaped, forming the floor of the braincase. The pointed cultriform process overlaps the sphenethmoid ventrally. The parasphenoid alary processes provide the floor for the otic capsules and form a slightly obtuse angle with the parasphenoid/cultriform process. The posteromedial process of the parasphenoid is truncate and almost reaches the margin of the foramen magnum. The parasphenoid alary processes are not in contact with the long medial ramus of the pterygoid. The massive neopalatine is fused posteromedially to the sphenethmoid. Postchoanal vomers are almost straight, clearly distinguishable, anterior to the neopalatine. Each vomer bears ca. seven teeth. The postchoanal process of the vomer is short and does not reach the neopalatine. The neopalatine does not connect to the inner surface of the maxilla.

*Maxillary arcade*. Both maxillae and premaxillae are dentate, apparently not exostosed. The premaxillae are separated medially, slightly inclined anteriorly. The alary processes of the premaxillae are broad and acuminate posteriorly, diverging from the midline. The alary processes are directed posterodorsally and have a moderate indentation along their anteromedial base. In dorsal view, the alary processes reach the level of the anteriormost margin of the maxillae. The palatine (medial) process of the premaxillae is long, acuminate, directed posterodorsally. The lateral process of the premaxillae is short, about half the size of the palatine process, acuminate and directed posterolaterally. The premaxillae do not contact the maxillae, although the maxillae slightly invest the premaxillae on both side. The maxillae are greatly expanded and the pars facialis is well developed, but not in contact with the maxillary process of the nasal. Anteriorly and in lateral view, the maxillae are squarish and about twice as high as posteriorly. The maxillae possess a highly reduced (almost non-existent) posterodorsal projection directed towards the zygomatic ramus of the squamosal with which it is not in contact. Posteriorly, the maxillae are in close contact with, but not fused to, the robust quadratojugals.

*Suspensory apparatus*. The triradiate pterygoid is moderately slender. The anterior ramus extends toward the braincase from the maxilla at the mid-orbit level and further extends against the anteroventral margin of the otic capsule via the long medial ramus. There is no contact between the otic capsule and the medial ramus. The posterior ramus is broad and flat, not in contact with the ventral ramus of the squamosal. The posterior ramus is slightly shorter than the medial. There is a short dorsal process on the anterior ramus projecting towards the zygomatic ramus of the squamosal, but not contacting it. The quadratojugal is robust, in contact with, but not fused to, the maxilla. The otic and ventral rami of the squamosal are well developed; the otic ramus does not extend over the lateral margin of the prootic. The ventral ramus of the squamosal is almost straight, narrow in lateral view, and extends from the quadratojugal to the posterodorsal margin of the orbit. The otic ramus is acuminate, smooth, and less than half the length of the narrower zygomatic ramus. The otic ramus bears a low crest laterally. The zygomatic ramus is long, slim, and acuminate in lateral profile, not in contact with the maxilla. The zygomatic rami do not appear exostosed.

*Mandible*. The dentary is long and moderately stout, posteriorly acuminate, fused to the small, arcuate mentomeckelian bone anteriorly. The mentomeckelians are separated medially. The dentary overlaps almost half of the angulosplenial length. The main component of the mandible is the angulosplenial, which is long and sigmoid, acuminate anteriorly. Anteriorly and in ventral view, the angulosplenial fails to extend near the maxilla-premaxilla articulation. The coronoid process is dorsomedial and well-developed, about half of the posterior ramus of the pterygoid.

**Fig. 9:**
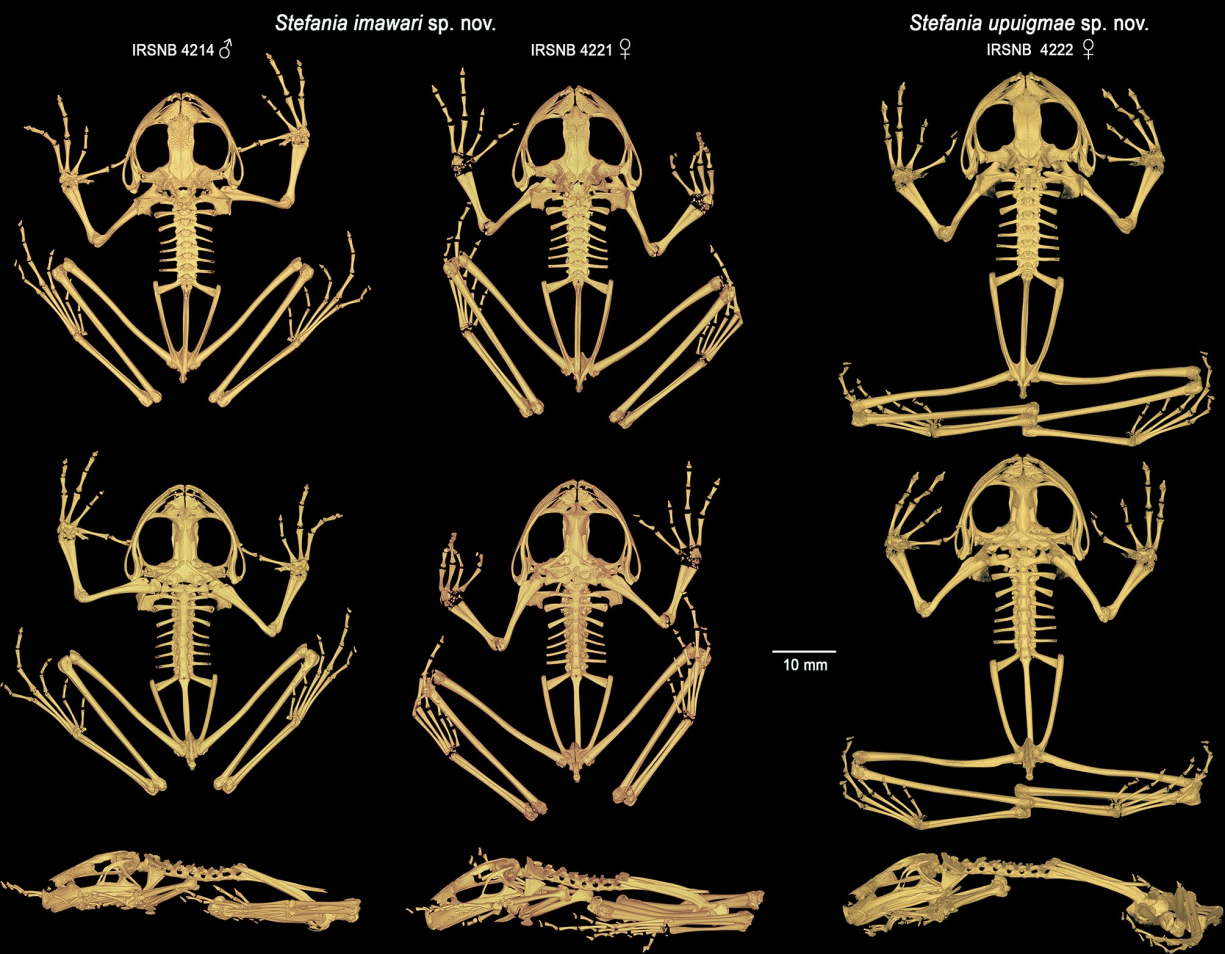
Three-dimensional models of the complete skeletons of one male paratype of *Stefania imawari* sp. nov. (IRSNB 4214) and the female holotype (IRSNB 4221), and of the female holotype of *S. upuigmae* sp. nov. (IRSNB 4222) based on µCT imagery, in dorsal, ventral and left lateral views

*Postcranium* (Fig. 9). The vertebral column is composed of eight non-imbricate, procoelous presacral vertebrae, sacrum, and urostyle. The atlantal cotylar arrangement corresponds to the Type I of Lynch [35]. Presacrals I–III expand dorsally. The transverse processes are moderately elongated, distally expanded on presacral II–IV. The length of the transverse processes is III > VIII = VII > VI > IV > V > II. The transverse processes of presacral II are directed roughly perpendicularly and ventrally to the medial axis; those of presacral III are directed posteroventrally; transverse processes of presacral IV to VII are directed posterodorsally; and those of presacral VIII are directed perpendicular and dorsally to the medial axis. Presence of small, paired calcified processes (likely calcified endolymphatic sacs; [36]) extending through the intervertebral foramina, but not, or barely, investing the ventral face of the transverse processes of the vertebrae. The sacral diapophyses are slightly flattened, expanded distally, and of a length similar to that of the transverse processes of presacral III. The sacral diapophyses are directed posterodorsally, with truncate distal borders and are not in contact with the ilia distally. The sacrum has a bicondylar articulation with the urostyle. The urostyle is similar in length to the presacral vertebral column, with its posterior tip upturned. Urostyle with a well-developed dorsal crest along most of its shaft. The crest originates anteriorly as a large, ossified tubercle and progressively decreases in height posteriorly. The pectoral girdle is arciferal. The clavicles are robust, flattened, arcuate, directed anteriorly, and moderately separated from each other medially; the clavicle appears to be barely in contact with the scapula and not attached to the coracoid. The posterior margin of the stout coracoid is weakly sigmoid, whereas the anterior margin is concave. The coracoids are separated and expanded medially, the concave glenoid and convex sternal ends are about equally expanded, almost three times as wide as the midshaft width of the bone. The cleithrum is dagger-shaped, the suprascapular cartilage appears to not be ossified. The head of the humerus is not ossified. A well-developed crista ventralis extends along the proximal half of the humerus. The crista lateralis is well visible in ventral (flexor) view, while the low crista medialis is only visible in lateral view. The capitulum and ulnar and radial condyles appear to be well developed but decalcified. The olecranon of the radio-ulna is round, the sulcus intermedius is indicated by a distinct groove; the epiphyses of the radius and ulna are decalcified, as well as all carpal elements and the prepollex (which is not visible). The finger phalangeal formula is standard (2–2–3–3), and the metacarpals increase in size in the following order: II, IV, I and III. The relative lengths of the fingers increase in size in the following order: II, I, IV, and III. The third metacarpal is too decalcified to detect the distal process (which is visible in the µCT-scanned paratype, see Variation). The distal phalanges are slightly curved downwards with pointed tips. The postsacral trunk region is relatively short and narrow. The articulation between the anterior end of the ilial shafts and the ventral side of the distal ends of the sacral transverse processes is of the sagittal-hinge type [37], usually characteristic of long-distance jumpers. The ilial shafts have large crests along almost their entire length, originating approximately at the level of the urostyle tubercle and terminating in a posterior prominence. The ilia are posteriorly in contact with the ischium but are apparently not fused to it. The pubis is decalcified; the acetabulum appears round and well developed. The femur is slightly shorter than the tibiofibula. The femur is weakly sigmoid and bears a posteroventral ridge on its proximal end. The sulcus intermedius of the tibiofibula is much less prominent than the sulcus intermedius of the radio-ulna. The astragalus and calcaneum are about two-thirds the size of the tibiofibula. These structures are widely separated at their midpoint and fused at their distal and proximal heads. Tarsal elements are highly decalcified, therefore difficult to appreciate. The toe phalangeal formula is standard (2–2–3–4–3), and the metatarsals increase in size in the following order: I, II, III, V, IV. The relative lengths of the toes increase in the same order. The phalangeal elements are decalcified, the ultimate phalange of the toes appears to be similar in shape and size to that of the fingers.

**Fig. 10:**
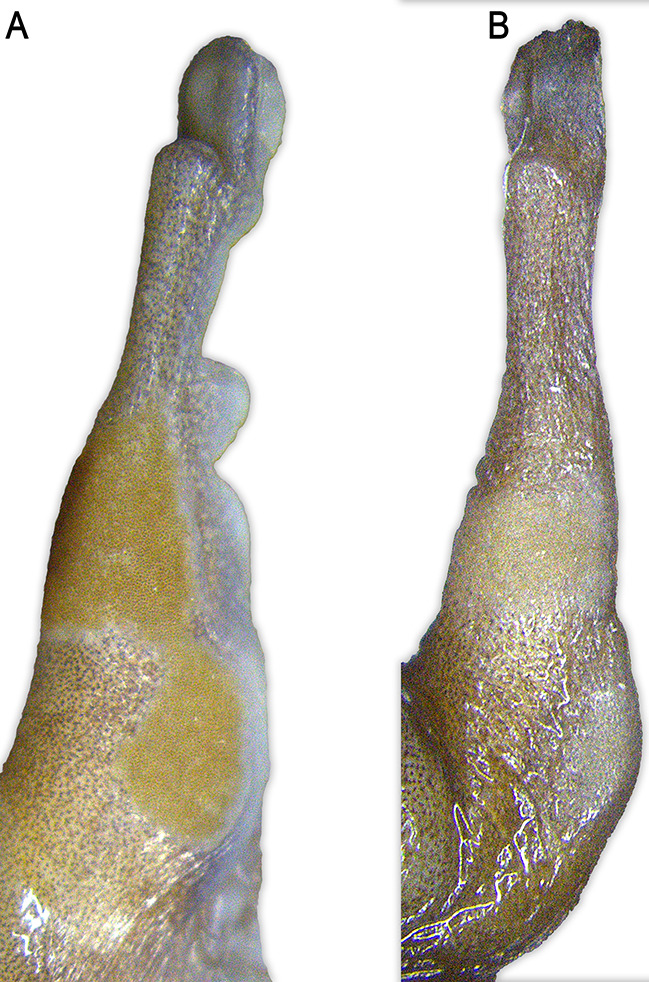
Male nuptial excrescences (thumb) in (**A**). *Stefania imawari* sp. nov. (IRSNB 4219) and (**B**). *S. lathropae* (IRSNB 4212). Photos by the author

**Variation**. As in many *Stefania* species, *S. imawari* sp. nov. is polychromatic (Fig. 7), and polychromatism does not seem to be related to sex. Adult individuals range from almost plain golden brown, chestnut brown, medium brown to dark greenish brown, with more or less extensive dark brown markings and/or creamy white/beige spots and stripes on the dorsal surfaces. A few faint light greenish brown chevrons are visible on the dorsum of a small immature female (Fig. 7B). Dorsal surface of thighs may be immaculate or bear conspicuous transverse bars. In life, a canthal dark brown/black stripe is present in all specimens examined (usually not visible in preservative); no specimen had a distinct interorbital bar. Iris faintly bicolour in all specimens examined, a faint blueish grey tint is rarely present. The dorsal skin condition (tuberculate) is similar across specimens examined, with no major difference detected. Males are smaller than females: 44.1–48.9 mm SVL in adult males (*n* = 5) vs. 48.5–55.4 mm SVL in adult females (*n* = 3). Head is slightly longer than wide, or slightly wider than long. The number of vomerine teeth ranges from three to seven. There is no significant difference in toe webbing between the sexes; toe webbing –for all adult males (*n* = 5) is II 1 ^3^/_4_–(3–3^+^) III (2–2^+^)–(3 ^1^/_3_–3 ^1^/_2_) IV (3 ^1^/_4_–3^+^)–(2^−^–2) V; toe webbing formula for all adult females (*n* = 3) is II (1 ½–1 ^3^/_4_)–3 III (2–2^+^)–(3 ^1^/_3_–3 ^1^/_2_) IV (3 ^1^/_4_–3^+^)–(2^−^–2) V. One female (IRSNB 4217) has basal webbing between the first and second toe (I 2–2 ^1^/_2_ II). All males examined have detectable nuptial excrescences (Fig. 10), and a slightly darker throat than females, especially along the lower lip and under the corner of the mouth (better appreciated in preservative). There is a small distal process on the third metacarpal in the µCT-scanned paratype, which has its extremities less decalcified than the holotype. The carpus is easier to appreciate in that specimen, and is composed of a radiale, ulnare, ossified prepollex element, element Y, distal carpal 2 and an element representing the fusion of distal carpals 3–5 (dcpl 3–5). Both ulnare and dcpl 3–5 display a lateral apophysis on their dorsal border. Tarsal elements are also easier to appreciate in the paratype, with the presence of two tarsals (distal tarsal 1 and an element representing the fusion of distal tarsals 2–3) at the base of Toes II–III. A small element Y and a short ossified prehallux element are also present, at the base of Toe I.

**Distribution and natural history**. *Stefania imawari* sp. nov. is only known from the type locality, i.e., the summit of Angasima-tepui between 2,122 and 2,154 m elevation (Figs. 1 and 2). The species may be present on Katuran-tepui, a neighbouring mountain referred as Angasima-tepui “W branch” in Gorzula and Señaris [34] in their account on *Stefania satelles*, but this should be confirmed by molecular data as the summits of Katuran-tepui and Angasima-tepui are presently well isolated from each other.

The summit of Angasima-tepui is a mix of exposed rocks and richly vegetated areas, described as “*low tepui-summit scrub and meadows on peat and rock*” by McDiarmid and Donnelly [9] (Fig. 2). The summit is fractured into a southern and a northern part by a wide canyon mostly covered by low-growing tepui forest.

Specimens of *Stefania imawari* sp. nov. were found active at night, mostly sitting on rocks or on the top of the bromeliad *Brocchinia hechtioides*. One female was found inside a *B. hechtioides*. A few specimens were on the ground on low vegetation at night, and three specimens were found hiding under rocks during the day. No female carrying eggs or juveniles was observed during a short stay on the summit of Angasima-tepui (11–16 May 2011, i.e., during the rainy season), but one female (IRSNB 4220, 55.4 mm SVL) retained a glutinous patch on the back (Fig. 7E).

### *Stefania upuigmae* sp. nov

*Stefania satelles* [in part] Señaris et al., 1997 [8]: 33–37.

*Stefania satelles* [in part] Gorzula and Señaris, 1999 [34]: 47.

*Stefania satelles* [in part] McDiarmid and Donnelly, 2005 [9]: 513, 521.

*Stefania satelles* [in part] Kok et al., 2016 [14]: 6.

*Stefania* sp. 5 Kok et al., 2017 [10]: 175–176.

**ZooBank registration**. urn: lsid: zoobank.org: act:973B0004-5714-4ECA-8D08-ADA1FBEBFCC8.

**Fig. 11:**
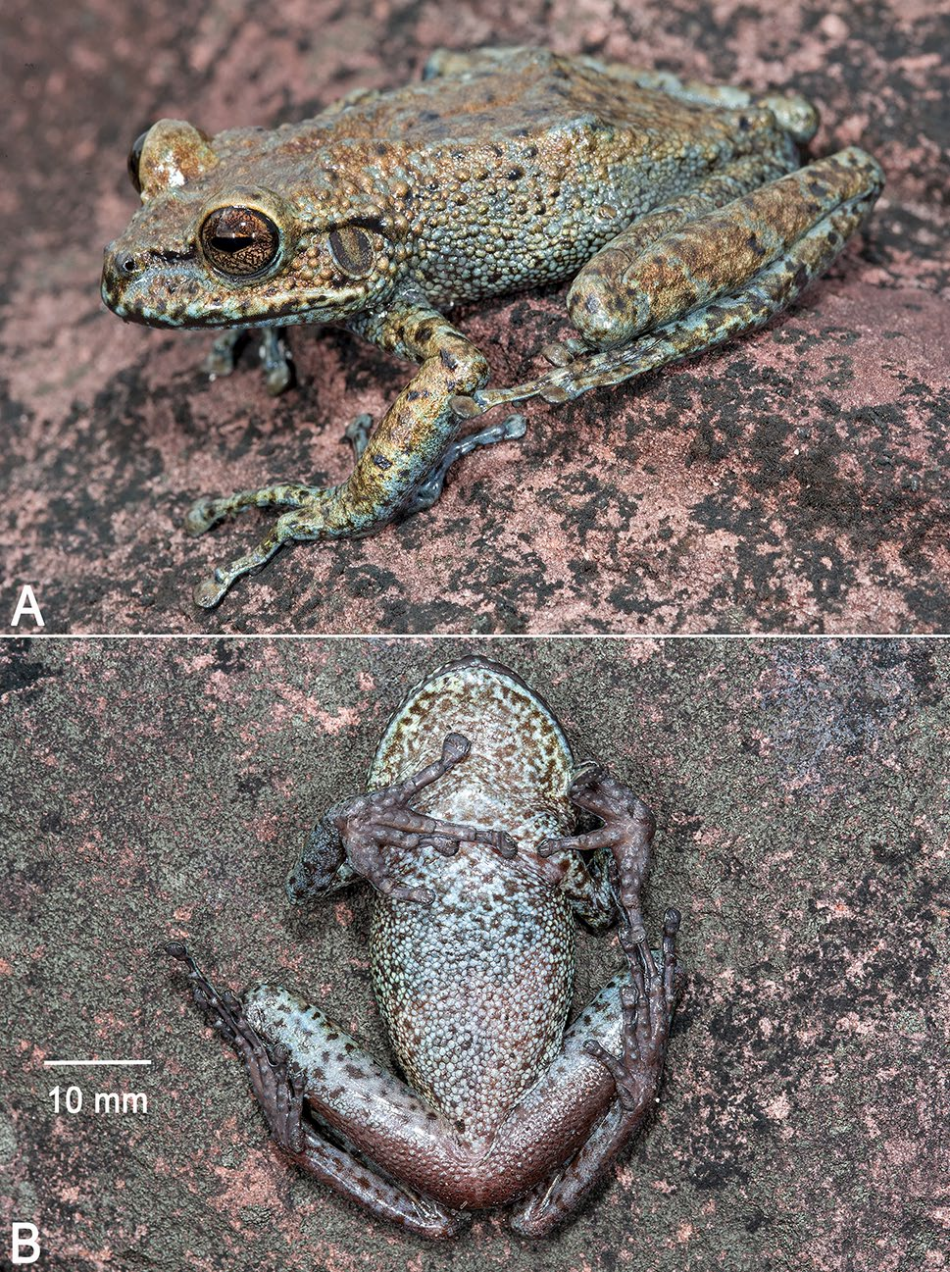
Holotype of *Stefania upuigmae* sp. nov. (IRSNB 4222, female, 53.1 mm SVL). (**A**). Dorsolateral view in life. (**B**). Ventral view of the specimen freshly euthanized. Photos by the author

**Fig. 12:**
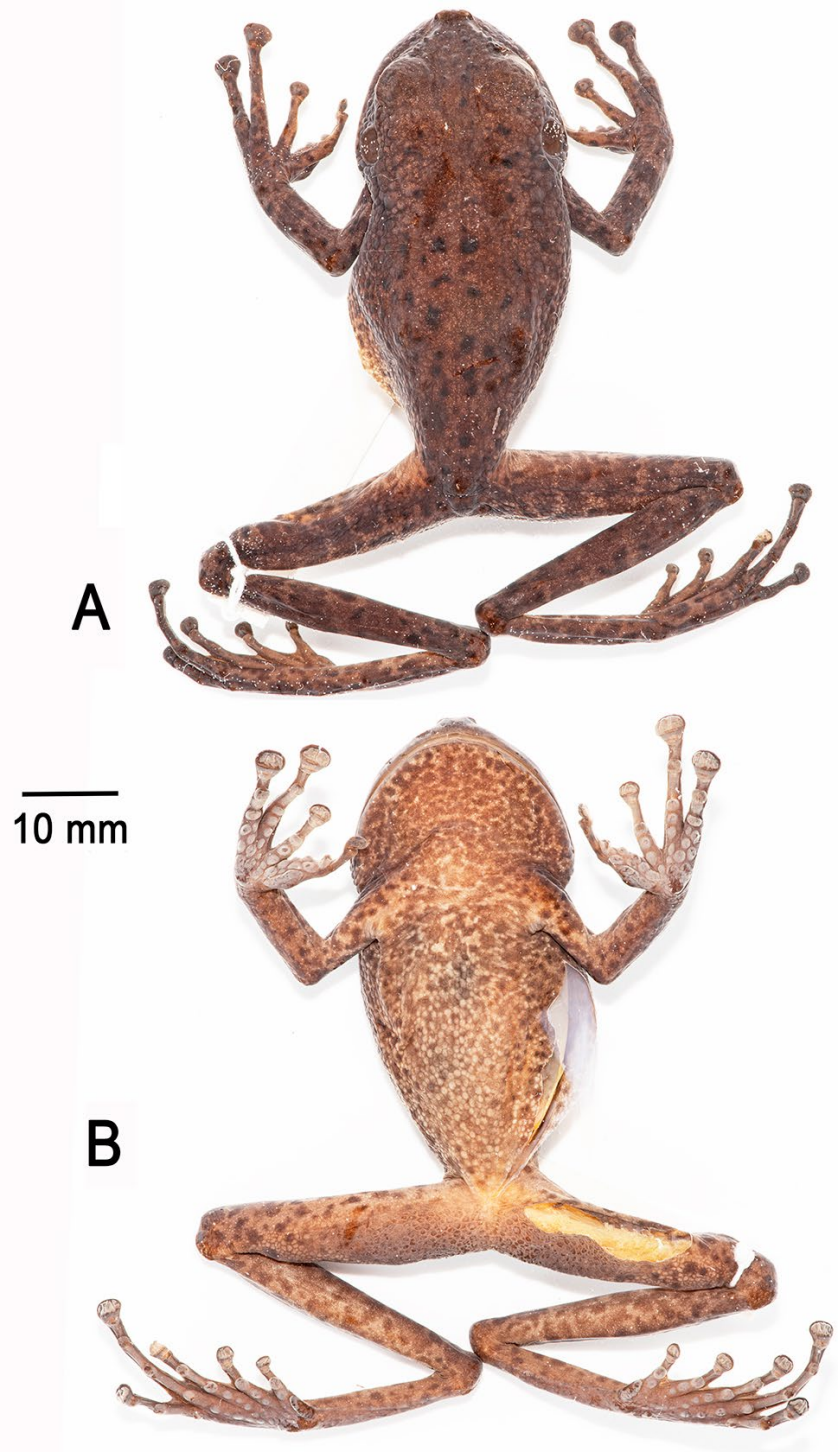
Holotype of *Stefania upuigmae* sp. nov. (IRSNB 4222, female, 53.1 mm SVL) after 12 years in ethanol preservative (**A**). Dorsal view. (**B**). Ventral view. Photos by the author

**Fig. 13:**
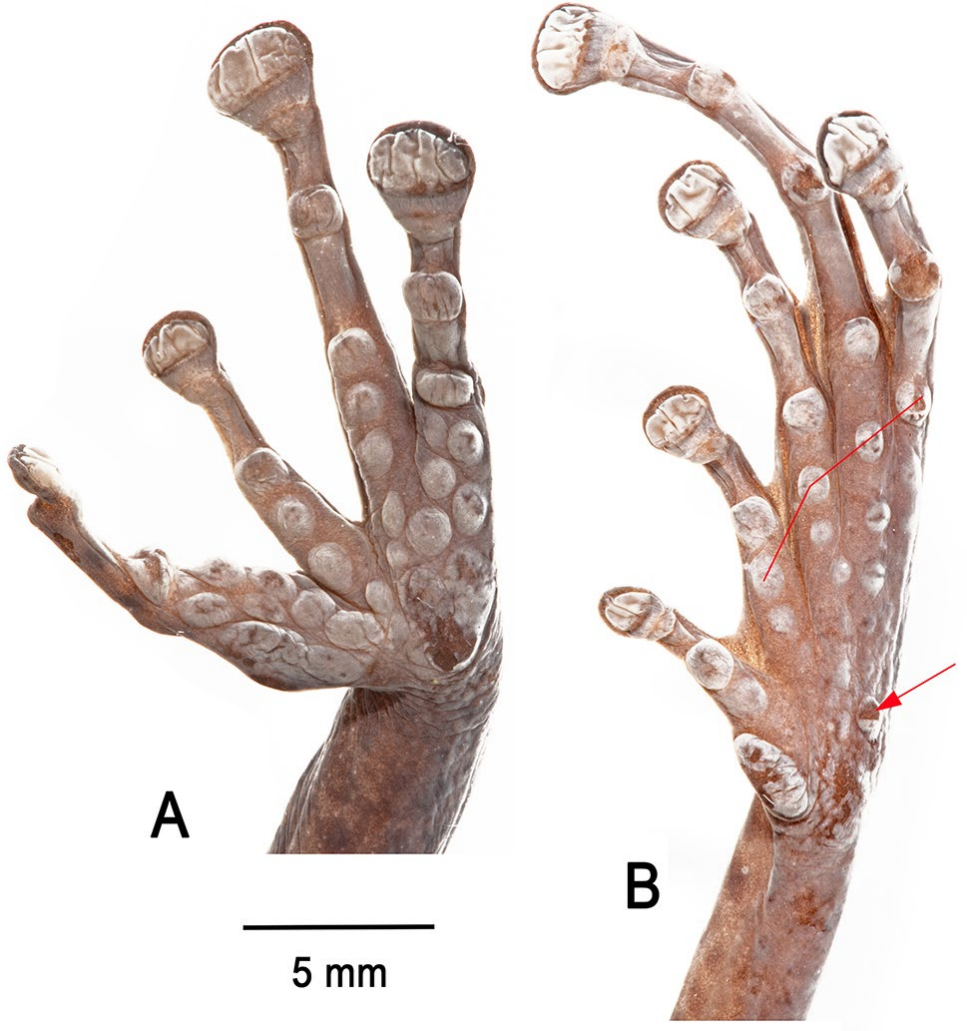
Palm (**A**) and sole (**B**) of the preserved holotype of *Stefania upuigmae* sp. nov. (IRSNB 4222, female, 53.1 mm SVL). The thin red line highlights a row of distinctly enlarged supernumerary plantar tubercles (absent in *S. imawari* sp. nov.; compare with Fig. 6). The red arrow highlights the distinctly enlarged outer metatarsal tubercle (not enlarged in *S. imawari* sp. nov.; compare with Fig. 6). Photos by the author

**Holotype**. IRSNB 4222 (field number PK3615, Figs. 11, 12 and 13), an adult female collected by Philippe J. R. Kok and Brad Wilson, 8 June 2012 at 11h30, summit of Upuigma-tepui, Bolívar State, Venezuela (5°05′11″N, 61°57′33″W; 2,134 m elevation).

**Etymology**. The specific epithet *upuigmae* is a noun in the genitive case and refers to the type locality, the summit of Upuigma-tepui, also locally known as “El Castillo”.

**Definition and diagnosis**. Based on the holotype female, *Stefania upuigmae* sp. nov. is characterized by the following morphological characters, the combination of which distinguishes it from all known congeners: (1) a small species of *Stefania*, SVL of the preserved adult holotype female 53.1 mm; (2) head not distinctly longer than wide, about as wide as long; (3) canthus rostralis angular, slightly concave in dorsal view (almost straight), tuberculate, 1–3 distinctly enlarged canthal tubercles that project dorsolaterally, canthal stripe present in life; (4) loreal region with a few low tubercles; (5) upper eyelid smooth; (6) frontoparietal ridges present but low and barely visible (both in life and in preservative); (7) frontoparietal crests barely developed (on cranium); (8) no distinct constriction of the frontoparietal bones at the level of the anterior epiotic eminence; (9) exostosis on the cranium highly reduced and barely visible; (10) premaxillae slightly projecting posteriorly in lateral view; 11) posterodorsal projection of maxilla absent; 12) maxillary process of the nasal not in contact with the maxilla; 13) horizontal length of tympanum more than 50% horizontal length of eye; 14) vomerine teeth 1–4; 15) toes basally webbed; 16) dorsal skin (in life) tuberculate; 17) ventral skin (in life) granular; 18) presence of a conspicuous/enlarged outer tarsal tubercle; 19) absence of multiple conspicuous dark brown bars on flanks and lips, absence of white dorsolateral stripes (in life); 20) in life, iris unicolour, medium brown/copper, with extensive dark brown reticulations.

*Stefania upuigmae* sp. nov. is further distinguished from the other described species in the *S. ginesi* clade as follows:

From *Stefania ginesi* principally by the colour of the iris (greyish blue in *S. ginesi* vs. medium brown/copper in *S. upuigmae* sp. nov.); by the size and number of tubercles on the dorsum, flanks, and limbs (distinctly larger and more numerous in *S. ginesi*); by a more angular canthus rostralis (less defined in *S*. ginesi); and by the size of tubercles on the canthus rostralis and upper eyelid (larger in *S. ginesi*).

From *Stefania imawari* sp. nov. principally by a heavily mottled belly (vs. no mottling in *S. imawari* sp. nov.); by a unicolour iris in life (vs. faintly bicolour in *S. imawari* sp. nov.); by the presence of enlarged supernumerary plantar tubercles and an enlarged outer metatarsal tubercle (not enlarged in *S. imawari* sp. nov.); by the size of the choanae (distinctly larger in *S. upuigmae*); and by the size of the odontophores and the number of vomerine teeth (odontophores distinctly shorter with much less teeth in the holotype of *S. upuigmae* sp. nov.).

From *Stefania lathropae* mostly by the absence of white dorsolateral stripes (always present, even if sometimes short, in *S. lathropae*). A notable potential osteological diagnostic character is the length of the postchoanal process of the vomer (long and reaching the neopalatine in *S. lathropae* vs. short in *S. upuigmae* sp. nov.).

From *Stefania satelles* mostly by the condition of the frontoparietal crests (moderately developed and distinctly projecting dorsolaterally in *S. satelles* vs. barely developed in *S. upuigmae* sp. nov.); and by the shape of the snout in lateral view (sloping from below the nostrils in *S. satelles* vs. sloping from the nostrils in *S. upuigmae* sp. nov.).

**Description of the holotype**. An adult female 53.1 mm SVL (Figs. 11, 12 and 13), in good condition except for long incisions made during tissue sampling (left thigh) and during the examination of internal organs (left side of the abdomen). Head slightly wider than long, wider than neck. Snout rounded in dorsal and lateral views, slightly mucronate in dorsal view, ca. 1.5 times the horizontal length of eye. Eye–nostril distance ca. 1.1 times the horizontal length of eye, more than three times the distance between the nostril and the tip of the snout. Canthus rostralis angular, slightly concave in dorsal view (almost straight), sloping; lips flared. Nostrils protuberant, directed anterolaterally. Internarial distance almost 70% of interorbital distance. Internarial region concave. Interorbital distance slightly shorter than upper eyelid width. Frontoparietal ridges are low and barely visible. Temporal region slightly bulged. Tympanum distinct, large, round, directed posterolaterally, 73% of horizontal length of eye, separated from eye by ca. 58% of horizontal length of eye. Supratympanic fold prominent, extending from posterior corner of eye to above insertion of forelimb, obscuring upper margin of tympanum.

Choanae large and oval. Vomerine processes transverse between choanae, not in contact, smaller than choanae, each bearing ca. 1–4 teeth. Tongue large, ovoid. Palpebral membrane not reticulated, upper rim with a light brown band.

Dorsal skin tuberculate, venter and posterior surface of thighs granular; a few low tubercles on shank. Upper eyelid mostly smooth, no enlarged tubercle on upper eyelid. Loreal region with a few low tubercles. Tympanic region strongly tuberculate. Canthus rostralis tuberculate, one canthal tubercle distinctly enlarged and projecting dorsolaterally. Cloacal opening directed posteriorly at upper level of thighs, presence of a short cloacal flap.

Thenar tubercle large, distinct, transversely oval; palmar tubercle distinct, about the same length as the thenar tubercle. Subarticular tubercles distinct, large, projecting, and single in appearance. Supernumerary tubercles numerous, large, and projecting (Fig. 13). Relative finger lengths II < I < IV < III; adpressed second finger barely reaches the first finger’s disc. Fingers with lateral fringes; narrow webbing between FIII-FIV, which are fused proximally. Finger discs large, transversely oval, more than two times wider than adjacent phalange on FIII and FIV, smallest and subequal in length on FI and FII, largest and subequal in length on FIII-IV. Largest disc width (FIII) 76% of horizontal length of tympanum.

Inner metatarsal tubercle large, oval, distinct; outer metatarsal tubercle distinct, round, about half the size of the inner metatarsal tubercle. Subarticular tubercles single, round, distinct. Supernumerary tubercles numerous, round, distinct, most of them similar in size, except for one distinctly enlarged supernumerary tubercle below each of the first subarticular tubercles (Fig. 13). Relative lengths of toes I < II < III < V < IV; adpressed fifth toe distinctly longer than third. Toes basally webbed, webs tapering to lateral fringes. Webbing formula I 2–2^+^ II 1 ^1^/_2_–3 III 2–3 ^1^/_2_ IV 3 ^1^/_3_–2 V. Toe discs oval, wider than adjacent phalange, largest toe disc on Toe IV, ca. 71% width of largest finger disc. Heels overlapping when hindlimbs are folded at right angles to sagittal body plane.

**Colour of holotype in life**. Dorsum light to medium brown with a few black and metallic greenish blue spots/markings (Fig. 11). Flanks speckled with black, dark brown, medium brown and metallic greenish blue, all spots corresponding to individual tubercles or grouping of a few tubercles. Anterodorsal aspect of upper and lower arm light greenish brown, with a few brown patches (very faint transverse bands) and some black/dark brown markings. Anterodorsal aspect of thigh and shank with ill-defined medium brown and metallic greenish blue transverse bands (the former much wider than the latter), with a few brown and dark brown spots. Hands metallic greenish blue speckled with dark brown/black; feet metallic greenish blue with ill-defined medium brown transverse markings/bars and some dark brown/black spots and markings. Top of head and upper eyelid medium brown, similar to dorsum, but without any dark brown/black spot. Canthal stripe black, almost reaching the nostril, short black supratympanic stripe present. Tympanum dark grey, almost black with a few tiny brown speckles, central portion of tympanum greenish brown; tympanum circled in brown and metallic greenish blue. Upper lip metallic greenish blue, with some scattered black and brown spots/markings; lower lip bluish grey with a few dark brown markings. Throat bluish grey, heavily mottled with dark brown. Thorax, venter, and underside of limbs bluish grey, spotted/mottled with dark brown and black. Belly mostly opaque (internal organs barely visible). Palms and soles dark pinkish grey. Iris unicolour, medium brown/copper, with extensive dark brown reticulations.

**Colour of holotype in preservative**. After almost 12 years in ethanol preservative (Fig. 12), the dorsal colouration turned dark/medium brown with black spots/markings; all metallic greenish blue markings disappeared. The ventral face turned creamy brown, and the medium brown mottling became even more conspicuous. Palms turned light grey, and soles are now dark greyish brown.

**Fig. 14:**
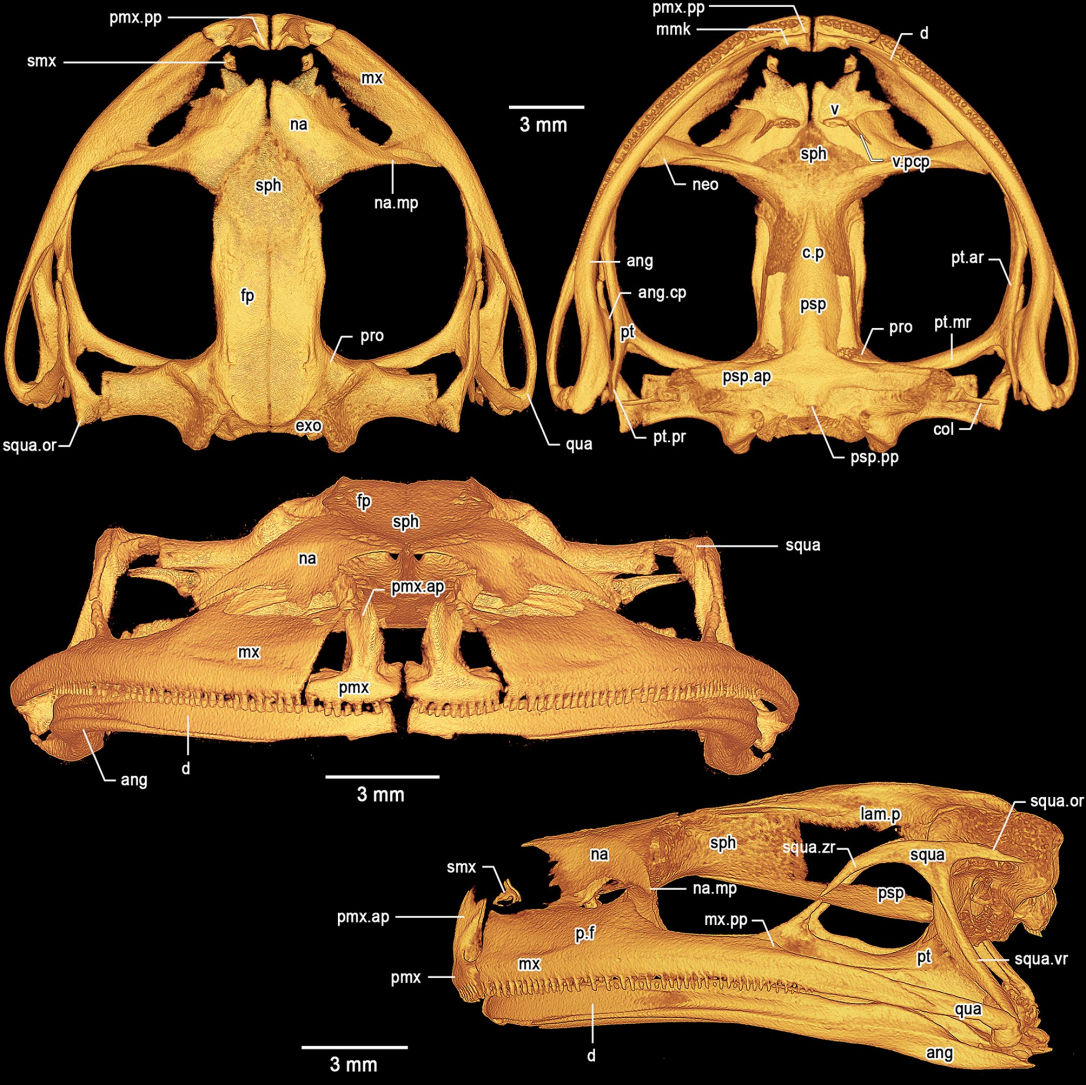
Three-dimensional model of the cranium of the female holotype of *Stefania upuigmae* sp. nov. (IRSNB 4222) based on µCT imagery. Top left: dorsal view. Top right: ventral view. Middle: frontal view. Bottom: left lateral view. Abbreviations: ang = angulosplenial, ang.cp = coronoid process of the angulosplenial, col = columella, c.p = cultriform process, d = dentary, exo = exoccipital, fp = frontoparietal, lam.p = lamina perpendicularis, mmk = mentomeckelian, mx = maxilla, mx.pp = posterodorsal projection of the maxilla, na = nasal, na.mp = maxillary process of the nasal, neo = neopalatine, p.f = pars facialis, pmx = premaxilla, pmx.ap = alary process of the premaxilla, pmx.lp = lateral process of the premaxilla, pmx.pp = palatine process of the premaxilla, pro = prootic, psp = parasphenoid, psp.ar = alary process of the parasphenoid, psp.pp = posteromedial process of the parasphenoid, pt = pterygoid, pt.ar = anterior ramus of the pterygoid, pt.mr = medial ramus of the pterygoid, pt.pr = posterior ramus of the pterygoid, qua = quadratojugal, smx = septomaxilla, sph = sphenethmoid, squa = squamosal, squa.or = otic ramus of the squamosal, squa.vr = ventral ramus of the squamosal, squa.zr = zygomatic ramus of the squamosal, v = vomer, v.pcp = postchoanal process of the vomer

**Osteology of holotype**.*Cranium* (Fig. 14). The skull is large, wider than long (longest width ca. 124% of medial length). The braincase appears mostly ossified; the sphenethmoid complex is dorsally slightly invested by the nasals. The exoccipital-prootic complex is well ossified and laterally overlapped by the otic ramus of the squamosal. The paired septomaxillae are well developed and lie dorsal to the palatine process and posterolaterally to the articulation between the maxilla and premaxilla. The columellae (stapes) are ossified, formed by the synostotic fusion of the long, thin pars media plectri (stylus) and the pars interna plectri (baseplate), which is curved.

*Dorsal investing bones*. The nasals are broad, not exostosed, not contacting medially. The posteromedial margins of the nasals slightly invest the sphenethmoid. The maxillary process of the nasal is long, acuminate, almost straight in dorsal view, not in contact with the maxilla. The frontoparietals are mostly not exostosed and completely roof the central braincase from the anterior level of the orbit to the level of the tectum synoticum posteriorly. Lamina perpendicularis poorly developed along the anterior orbital margin of the frontoparietal, expanding posteriorly. Posteriorly, the frontoparietal slightly expands dorsally to form a very low frontoparietal crest, which does not bear any conspicuous exostosis.

*Ventral investing and palatal bones*. The parasphenoid is nib-shaped, forming the floor of the braincase. The pointed cultriform process overlaps the sphenethmoid ventrally. The parasphenoid alary processes provide the floor for the otic capsules and form a slightly obtuse angle with the parasphenoid/cultriform process. The posteromedial process of the parasphenoid is truncate and almost reaches the margin of the foramen magnum. The parasphenoid alary processes are not in contact with the long medial ramus of the pterygoid. The massive neopalatine is fused posteromedially to the sphenethmoid. Postchoanal vomers are straight, clearly distinguishable, anterior to the neopalatine. Each vomer bears ca. 1–4 teeth. The postchoanal process of the vomer is moderately long but does not reach the neopalatine. The neopalatine does not connect to the inner surface of the maxilla.

*Maxillary arcade*. Both maxillae and premaxillae are dentate, apparently not exostosed. The premaxillae are separated medially, slightly inclined anteriorly. The alary processes of the premaxillae are broad and acuminate posteriorly, diverging from the midline. The alary processes are directed posterodorsally and have a moderate indentation along their anteromedial base. In dorsal view, the alary processes reach the level of the anteriormost margin of the maxillae. The palatine (medial) process of the premaxillae is moderately long, acuminate, directed posterodorsally. The lateral process of the premaxillae is short, about half the size of the palatine process, acuminate and directed posterolaterally. The premaxillae do not contact the maxillae, and the premaxillae are not invested by the maxillae. The maxillae are greatly expanded and the pars facialis is well developed but not in contact with the maxillary process of the nasal. Anteriorly and in lateral view, the maxillae are squarish and about twice as high as posteriorly. The maxillae possess a highly reduced (almost non-existent) posterodorsal projection directed towards the zygomatic ramus of the squamosal with which it is not in contact. Posteriorly, the maxillae are partly fused to the robust quadratojugals.

*Suspensory apparatus*. The triradiate pterygoid is moderately slender. The anterior ramus extends toward the braincase from the maxilla close to the anterior edge of orbit and further extends against the anteroventral margin of the otic capsule via the long medial ramus. There is no contact between the otic capsule and the medial ramus. The posterior ramus is broad and flat, not in contact with the ventral ramus of the squamosal. The posterior ramus is slightly shorter than the medial one. There is a medium dorsal process on the anterior ramus projecting towards the zygomatic ramus of the squamosal, but not contacting it, although these structures are very close to each other. The quadratojugal is robust, partly fused to the maxilla. The otic and ventral rami of the squamosal are well developed; the otic ramus does not extend over the lateral margin of the prootic. The ventral ramus of the squamosal is slightly curved, narrow in lateral view, and extends from the quadratojugal to the posterodorsal margin of the orbit. The otic ramus is acuminate, smooth, and only slightly shorter than the narrower zygomatic ramus. The otic ramus bears a low crest laterally. The zygomatic ramus is moderately long, slim, and acuminate in lateral profile, not in contact with the maxilla. The zygomatic rami do not appear exostosed.

*Mandible*. The dentary is long and moderately stout, posteriorly acuminate, fused to the small, arcuate mentomeckelian bone anteriorly. The mentomeckelians are separated medially. The dentary overlaps about 1/3 of the angulosplenial length. The main component of the mandible is the angulosplenial, which is long and sigmoid, acuminate anteriorly. Anteriorly and in ventral view, the angulosplenial fails to extend near the maxilla-premaxilla articulation. The coronoid process is dorsomedial and well-developed, about half of the posterior ramus of the pterygoid.

*Postcranium* (Fig. 9). The vertebral column is composed of eight non-imbricate, procoelous presacral vertebrae, sacrum, and urostyle. The atlantal cotylar arrangement corresponds to the Type I of Lynch [35]. Presacrals I–III expand dorsally. The transverse processes are moderately elongated, distally expanded on presacral II–IV. The length of the transverse processes is III > VIII = VII > VI > IV > V > II. The transverse processes of presacral II are directed roughly perpendicularly and ventrally to the medial axis; those of presacral III are directed posteroventrally; transverse processes of presacral IV to VI are directed posterodorsally; and those of presacrals VII and VIII are directed perpendicular and dorsally to the medial axis. Presence of small, paired calcified processes (likely calcified endolymphatic sacs; [36]) extending through the intervertebral foramina, but not, or barely, investing the ventral face of the transverse processes of the vertebrae. The sacral diapophyses are slightly flattened, expanded distally and of similar length to the transverse processes of presacral III. The sacral diapophyses are directed posterodorsally, with truncate distal borders and are not in contact with the ilia distally. The sacrum has a bicondylar articulation with the urostyle. The urostyle is similar in length to the presacral vertebral column, with its posterior tip upturned. It bears a well-developed dorsal crest along ca. 2/3 of its shaft. The crest starts anteriorly as a large, ossified tubercle and progressively decreases in height in the caudal direction. The pectoral girdle is arciferal. The clavicles are robust, flattened, arcuate, directed anteriorly, and moderately separated from one another medially; the clavicle appears to be barely in contact with the scapula and not attached to the coracoid. The posterior margin of the stout coracoid is weakly sigmoid, whereas the anterior margin is concave. The coracoids are separated and expanded medially, the concave glenoid and straight sternal ends are about equally expanded, almost two times as wide as the midshaft width of the bone. The cleithrum is a dagger-shaped element, the suprascapular cartilage appears to not be ossified. The head of the humerus is not ossified. A well-developed crista ventralis extends along the proximal half of the bone. The crista lateralis is well visible in ventral (flexor) view, while the low crista medialis is only visible in lateral view. The capitulum and ulnar and radial condyles appear to be well developed, but decalcified. The olecranon of the radio-ulna is round, the sulcus intermedius is indicated by a distinct groove; the epiphyses of the radius and ulna are decalcified, as well as all carpal elements and the prepollex. The finger phalangeal formula is standard (2–2–3–3), and the metacarpals increase in size in the following order: II, IV, I and III. The relative lengths of the fingers increase in size in the following order: II, I, IV, and III. The third metacarpal is too decalcified to properly appreciate the distal process. The distal phalanges are slightly curved downwards with a pointed tip. The postsacral trunk region is rather long and narrow. The articulation between the anterior end of the ilial shafts and the ventral side of the distal ends of the sacral transverse processes is of the sagittal-hinge type [37], usually characteristic of long-distance jumpers. The ilial shafts have large crests along almost their full length, originating approximately at the level of the urostyle tubercle and terminating in a posterior prominence. The ilia are posteriorly in close contact with the ischium but are apparently not fused to it. The pubis is decalcified; the acetabulum appears round and well developed. The femur is slightly shorter than the tibiofibula. The femur is weakly sigmoid and bears a posteroventral ridge on its proximal end. The sulcus intermedius of the tibiofibula is much less prominent than the sulcus intermedius of the radio-ulna. The astragalus and calcaneum are about two-thirds the size of the tibiofibula. These structures are widely separated at their midpoint and fused at their distal and proximal heads. Tarsal elements are highly decalcified, therefore difficult to appreciate. The toe phalangeal formula is standard (2–2–3–4–3), and the metatarsals increase in size in the following order: I, II, III, V, IV. The relative lengths of the toes increase in the same order. The phalangeal elements are decalcified, the ultimate phalange of the toes appears to be similar in shape and size to that of the fingers.

**Distribution and natural history**. *Stefania upuigmae* sp. nov. is only known from the type locality, i.e., the summit of Upuigma-tepui at 2,134 m elevation (Figs. 1 and 2). The summit of Upuigma-tepui is a mix of exposed rocks and richly vegetated areas, described as “*low tepui-summit scrub and meadows on peat and rock*” by McDiarmid and Donnelly [9] (Fig. 2). The summit is much less fractured than that of Angasima-tepui but is rugged with some deep cracks and narrow canyons (Fig. 2).

The female holotype of *Stefania upuigmae* sp. nov. was found during the day jumping in low vegetation, likely disturbed by the collectors. The specimen was not carrying eggs or juveniles at the date of collection, which corresponds to the rainy season.

It must be noted that two *Stefania* specimens from Upuigma-tepui are presumably housed in the Museo de Historia Natural La Salle, Caracas, Venezuela under the name *S. satelles* [8]. I was unable to examine these specimens, but they most likely represent *S. upuigmae*.

## Discussion

According to Kok et al. [10], the ancestor of *Stefania imawari* sp. nov. (previously reported as “*S*. sp. 3”) diverged from the ancestor of the clade *S. upuigmae* sp. nov. (previously reported as “*S*. sp. 5”) + *S*. sp. 4 during the Pliocene, ca. 4 Ma, probably by vicariance [10]. One can assume that this corresponds to the period Angasima-tepui disconnected and became isolated from what is now Upuigma-tepui and the southeastern part of the Chimantá Massif. The relatively poor morphological differentiation among species in that clade suggests homoplasy or symplesiomorphy, as also observed among tepui summit species in the *S. riveroi* clade from the Eastern Tepui Chain [28]. As mentioned elsewhere [e.g., 14], it is worth pointing out that the sister species of *S. upuigmae* sp. nov. (*S*. sp. 4, still undescribed) was previously confused with *S. ginesi* (e.g., [8, 34]) and not with *S. satelles* as all other members of that subclade (subclade B in Fig. 3). This highlights again how external phenotype alone can be misleading when assessing species diversity in the genus *Stefania*, especially given the high level of colour polymorphism observed within most species.

**Fig. 15:**
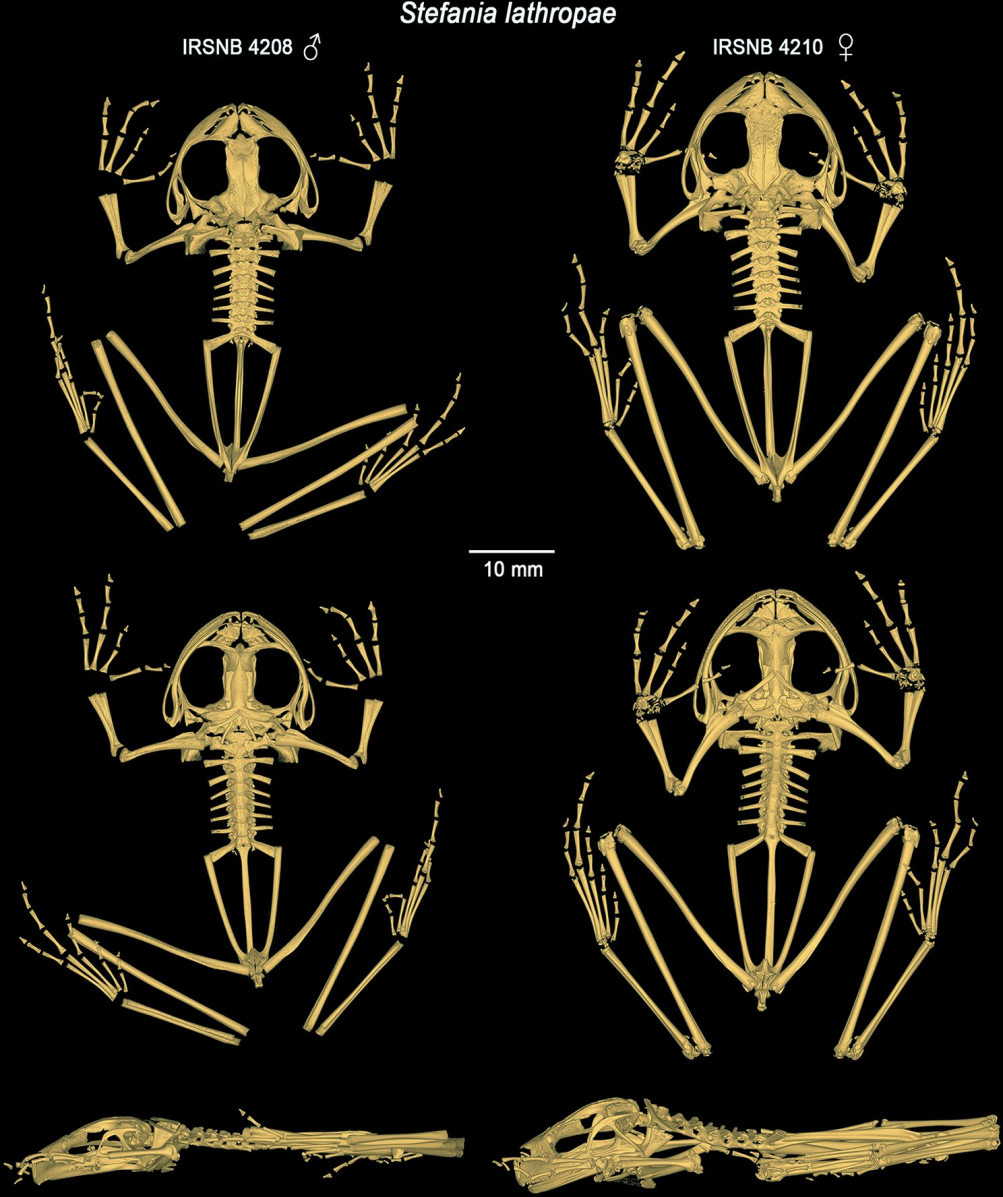
Three-dimensional models of the complete skeletons of the male holotype (IRSNB 4208) and one female paratype of *Stefania lathropae* (IRSNB 4210) based on µCT imagery, in dorsal, ventral and left lateral views

Osteological characters are helpful to discriminate among major clades within the genus *Stefania*, but are overall conserved among closely related species, and thus less conclusive for species recognition, especially across tepui summit populations, possibly also due to homoplasy/symplesiomorphy. The present study provides the first detailed imagery of the full skeleton of *S. lathropae* (Fig. 15; skull described in [15]), a closely related species, without showing any unambiguous difference with *S. imawari* sp. nov. and *S. upuigmae* sp. nov.

In accordance with criteria B1 and B2a, b [38], i.e., an extent of occurrence < 100 km^2^, an area of occupancy < 10 km^2^, a single known location, and projected decline—due to increasing anthropogenic fires in the region and effects of climate change [11, 39]—both *Stefania imawari* sp. nov. and *S. upuigmae* sp. nov. should be listed as Critically Endangered. Anthropogenic fires are reported as a major threat to tepui summit species, especially when their locality of occurrence is small (less than 3 km^2^ in *Stefania imawari* sp. nov. and *S. upuigmae* sp. nov.). In 1967 Mayr and Phelps [40] already noted that periodic burnings by residents left only sparse vegetation on most of the slopes of Upuigma-tepui, affecting the local bird fauna (see also [39]). The savannah patches visible in the photograph in Fig. 1 are all caused by anthropogenic fires.

## Conclusions

This study brings the number of described *Stefania* species to 22, making it the largest hemiphractid genus after *Gastrotheca* (which currently includes 77 species [1]). At least eight species still need to be described in the genus (see [10]; R. MacCulloch, pers. comm.). *Stefania imawari* sp. nov. and *S. upuigmae* sp. nov. are microendemic and morphologically/taxonomically cryptic species belonging to the *S. ginesi* clade. Described members of the *S. ginesi* clade are exclusively found on tepui summits between 2,115 and 2,580 m elevation in the Chimantá Massif and peripheral tepuis in the Bolívar State of Venezuela. Both new species are considered critically endangered according to IUCN criteria, mostly due to extremely small occurrence area (less than 3 km^2^). The population sizes needed to maintain population fitness on such small and isolated tepui summits are unknown. The likely complex population dynamics of isolated tepui summit species should be addressed in further research.

## Data Availability

Further information and requests for additional resources should be directed to and will be fulfilled by the author. This article has been registered in ZooBank with the life science identifier urn: lsid: zoobank.org: pub:255D8D9A-0B53-4E0E-87EA-01BE6139787F.

## Appendix – List of additional museum specimens examined

Institution acronyms are as follows: AMNH = American Museum of Natural History, New York, USA; CPI = Coastal Plains Institute and Land Conservancy, Tallahassee, USA; IRSNB = Royal Belgian Institute of Natural Sciences, Brussels, Belgium; MBUCV = Universidad Central De Venezuela, Caracas, Venezuela; MHNLS = Museo De Historia Natural La Salle, Caracas, Venezuela; ROM = Royal Ontario Museum, Toronto, Canada

### *Stefania ginesi* clade

*Stefania ginesi* (*n* = 2): IRSNB 16736–16737 (including µCT scans), summit of Chimantá-tepui, Bolívar State, Venezuela.

*Stefania lathropae* (*n* = 5): IRSNB 4208 (holotype; including µCT scans), IRSNB 4209, IRSNB 4210 (including µCT scans), IRSNB 4211–4212, summit of Murisipán-tepui, Bolívar State, Venezuela.

*Stefania satelles* (*n* = 7): MHNLS 10433 (holotype), IRSNB 16728 (including µCT scans), IRSNB 16729, IRSNB 19015 (including µCT scans), 19016–19018, summit of Aprada-tepui, Bolívar State, Venezuela.

### *Stefania riveroi* clade

*Stefania ayangannae* (*n* = 45): CPI 10473–10474, CPI 10656–10657 (including µCT scans), CPI 10818–10830, CPI 10911, CPI 10986–10992, CPI 11030–1,032, CPI 11040–11041, CPI 11070–11085, Mount Wokomung, Cuyuni-Mazaruni, Guyana.

*Stefania coxi* (*n* = 3): ROM 39479–39480 (paratypes, including µCT scans), Mount Ayanganna, Cuyuni-Mazaruni, Guyana; CPI 11093, Mount Wokomung, Cuyuni-Mazaruni, Guyana.

*Stefania maccullochi* (*n* = 18): NHMUK 2023.3184 (holotype; including µCT scans), NHMUK 2023.3185–3194, ROM 60548–554, summit of Wei-Assipu-tepui, Cuyuni-Mazaruni, Guyana.

*Stefania riveroi* (*n* = 24): MHNLS 10413 (holotype), MHNLS 10414–10416 (paratypes), MHNLS 11160 (paratype), IRSNB 15703, IRSNB 15715–15726, IRSNB 15727 (including µCT scans), IRSNB 15728–15729, IRSNB 15730 (including µCT scans), IRSNB 15740–15741, summit of Yuruaní-tepui, Bolívar State, Venezuela.

### *Stefania evansi* clade

*Stefania evansi* (*n* = 1): IRSNB 16738 (including µCT scans), Pakatau Creek, Potaro-Siparuni, Guyana.

*Stefania scalae* (*n* = 6): IRSNB 15674, IRSNB 15693, IRSNB 15694 (including µCT scans), La Escalera, Bolívar State, Venezuela; IRSNB 16724, El Danto, Bolívar State, Venezuela; CPI 10933 and CPI 10943 (including µCT scans), Kamarang Great Falls, Cuyuni-Mazaruni, Guyana.

### *Stefania woodleyi* clade

*Stefania woodleyi* (*n* = 3): IRSNB 13799, IRSNB 13800 (including µCT scans), IRSNB 13805 (including µCT scans), Kaieteur National Park, Potaro-Siparuni, Guyana.

*Stefania roraimae* (*n* = 5): IRSNB 15872, IRSNB 15873–15874 (including µCT scans), IRSNB 15875, IRSNB 15876 (including µCT scans), slopes of Maringma-tepui, Cuyuni-Mazaruni, Guyana.

### Incertae sedis

*Stefania breweri* (*n* = 1): MBUCV 6574 (holotype), summit of Cerro Autana, Amazonas State, Venezuela.

*Stefania goini* (*n* = 1): AMNH 23193 (holotype), Mount Duida, Amazonas State, Venezuela.

*Stefania oculosa* (*n* = 1): MHNLS 12961 (holotype), Jaua-tepui, Bolívar State, Venezuela.

*Stefania percristata* (*n* = 1): MHNLS 12952 (holotype), Jaua-tepui, Bolívar State, Venezuela.

*Stefania schuberti* (*n* = 3): MHNLS 12917 (holotype), IRSNB 16732 (including µCT scans), IRSNB 16733, Auyán-tepui, Bolívar State, Venezuela.

*Stefania tamacuarina* (*n* = 2): AMNH 131428 (holotype), MBUCV 6448 (paratype), Pico Tamacuari, Amazonas State, Venezuela.

## Abbreviations

ang: Angulosplenial
ang.cp: Coronoid process of the angulosplenial
col: Columella
c.p: Cultriform process
d: Dentary
dcpl: Distal carpal
exo: Exoccipital
FIII: Finger III
FIV: Finger IV
fp: Frontoparietal
lam.p: Lamina perpendicularis
ma: Million years ago
mmk: Mentomeckelian
mx: Maxilla
mx.pp: Posterodorsal projection of the maxilla
na: Nasal
na.mp: Maxillary process of the nasal
neo: Neopalatine
p.f: Pars facialis
pmx: Premaxilla
pmx.ap: Alary process of the premaxilla
pmx.lp: Lateral process of the premaxilla
pmx.pp: Palatine process of the premaxilla
pro: Prootic
psp: Parasphenoid
psp.ar: Alary process of the parasphenoid
psp.pp: Posteromedial process of the parasphenoid
pt: Pterygoid
pt.ar: Anterior ramus of the pterygoid
pt.mr: Medial ramus of the pterygoid
pt.pr: Posterior ramus of the pterygoid
qua: Quadratojugal
rRNA: Ribosomal RNA
smx: Septomaxilla
sph: Sphenethmoid
squa: Squamosal
squa.or: Otic ramus of the squamosal
squa.vr: Ventral ramus of the squamosal
squa.zr: Zygomatic ramus of the squamosal
SVL: Snout-vent length
TI: Toe I
TII: Toe II
v: Vomer
